# Polysaccharide-Based Composite Hydrogels as Sustainable Materials for Removal of Pollutants from Wastewater

**DOI:** 10.3390/molecules27238574

**Published:** 2022-12-05

**Authors:** Claudiu-Augustin Ghiorghita, Maria Valentina Dinu, Maria Marinela Lazar, Ecaterina Stela Dragan

**Affiliations:** Department of Functional Polymers, “Petru Poni” Institute of Macromolecular Chemistry, 700487 Iași, Romania

**Keywords:** sustainable development, polysaccharides, hydrogels, adsorption, wastewater treatment

## Abstract

Nowadays, pollution has become the main bottleneck towards sustainable technological development due to its detrimental implications in human and ecosystem health. Removal of pollutants from the surrounding environment is a hot research area worldwide; diverse technologies and materials are being continuously developed. To this end, bio-based composite hydrogels as sorbents have received extensive attention in recent years because of advantages such as high adsorptive capacity, controllable mechanical properties, cost effectiveness, and potential for upscaling in continuous flow installations. In this review, we aim to provide an up-to-date analysis of the literature on recent accomplishments in the design of polysaccharide-based composite hydrogels for removal of heavy metal ions, dyes, and oxyanions from wastewater. The correlation between the constituent polysaccharides (chitosan, cellulose, alginate, starch, pectin, pullulan, xanthan, salecan, etc.), engineered composition (presence of other organic and/or inorganic components), and sorption conditions on the removal performance of addressed pollutants will be carefully scrutinized. Particular attention will be paid to the sustainability aspects in the selected studies, particularly to composite selectivity and reusability, as well as to their use in fixed-bed columns and real wastewater applications.

## 1. Introduction

Improving the quality of water for human consumption/sanitation and/or domestic/industrial use is one of the 17 goals established by the United Nations for achieving sustainable development [1]. This goal was defined in the context of dramatic degradation of fresh water sources all over the world in recent decades, mainly driven by the growing human population and increase in anthropogenic activity [2,3]. Discharge of pollutants into surface water bodies has affected ecosystems, and ultimately created deleterious effects on all land-based life, including humankind [4]. According to their nature, pollutants can be classified as biological (pathogenic microorganisms), organic (dyes, oils, pharmaceuticals, plastics, pesticides/herbicides, etc.), and inorganic (heavy metal ions (HMIs), colloidal particles, etc.) [5]. Many of these pollutants are not biodegradable and, consequently, tend to accumulate in living organisms, causing different illnesses depending on their toxicological fingerprint [6,7].

**Figure 1:**
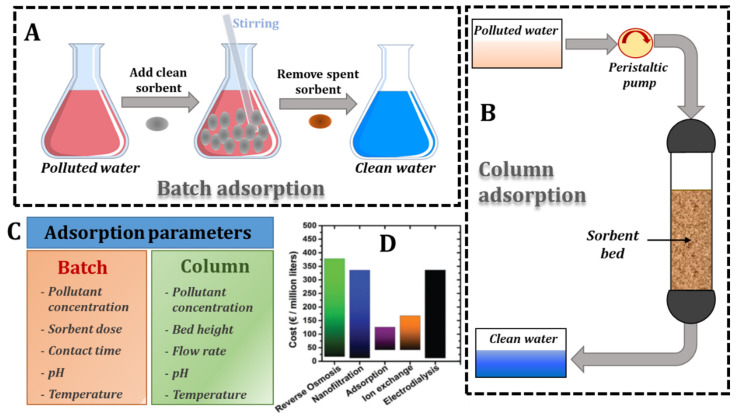
Illustrations of (**A**) batch and (**B**) column adsorption processes. (**C**) Parameters that affect the batch and column adsorption processes. (**D**) Cost per volume of treated water for various technologies applied in wastewater treatment (Reprinted with permission from Ref. [12]. Copyright 2019, Royal Society of Chemistry).

Nowadays, significant efforts are being undertaken to develop/improve different technologies (including coagulation/flocculation, bioremediation, oxidation, membrane filtration, and adsorption) to remove pollutants from wastewater and restore their quality, each presenting characteristic advantages and limitations [8,9,10,11,12,13]. A general issue with the successful implementation of the above listed technologies is related to the sustainability of engineered materials, and should address the following criteria: (i) have low fabrication costs, (ii) be adaptable to different characteristics of real wastewater, (iii) be resilient to long utilization times, and (iv) be environmentally benign [12]. Additionally, sustainability can also be strongly impacted by the technical and energy requirements for operation in pilot and/or industrial installations. Among the available wastewater treatment technologies, adsorption has been highlighted as the most sustainable one, mainly because it can be conducted with a minimal energy input and allows nearly complete water recovery [14]. From the operational standpoint, it can be successfully performed in static (batch) (Figure 1A) or in dynamic (column) (Figure 1B) modes, and the sorbents performance is correlated with the different parameters depicted in Figure 1C. Although stochastic considering the complementary characteristics of different technologies, the plethora of engineered materials, and the range of operating parameters, Bolisetty et al. appraised adsorption as the most cost-effective wastewater treatment method, with the lowest cost (in €) per millions of liters of produced water (Figure 1D) [12].

Driven by many salient advantages including natural abundance, renewable sources, facile extraction/purification, and low cost, polysaccharides have become increasingly attractive lately as building blocks to develop innovative and efficient sorbents for water purification [15,16,17]. The wealth and diversity of functional groups (-OH, -NH_2_, -COOH, -SO_3_H) in their structure promotes not only a high sorption performance, but also allows the engineering of structured materials with various compositions, geometries, and internal morphologies. In this context, composite hydrogels obtained using different polysaccharides, synthetic polymers and/or nanofillers (clays, zeolites, iron oxides, carbon nanotubes (CNT), graphene, graphene oxide (GO), metal organic frameworks (MOFs), etc.) are intensely developed worldwide as a means to address the sustainability criteria related to adaptability and resilience. They exhibit improved mechanical properties and elasticity [18,19], controlled porosity [20], and chemical stability [21], which render them more suitable to be used for long wear times in harsh environmental conditions.

**Figure 2:**
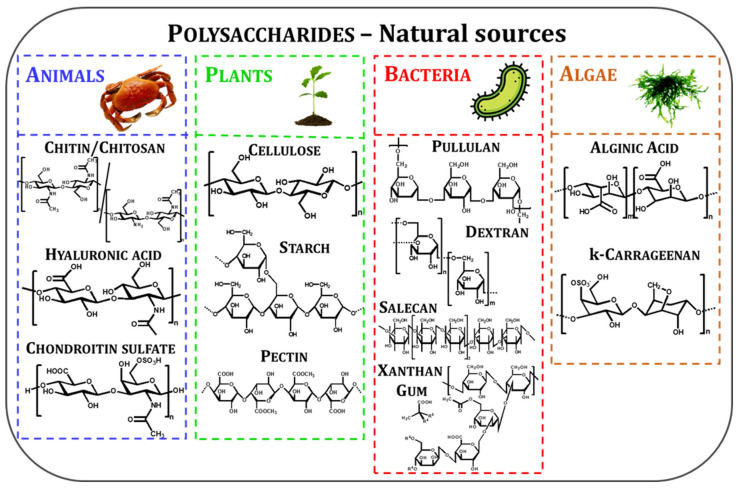
Chemical structures of a selection of polysaccharides, classified according to their natural sources.

- Polysaccharides are ubiquitously found in nature, fulfilling specific functions (i.e., structural support and energy storage) in the organisms that produce them. According to their origin (Figure 2), polysaccharides can be classified as follows [22]:
- *animal polysaccharides*: chitin (CT)/chitosan (CS), hyaluronic acid (HA), chondroitin sulfate (CRS), etc.;
- *plant polysaccharides*: cellulose (Cel), starch (St), pectin (Pec), etc.;
- *bacterial polysaccharides*: pullulan (Pul), dextran (Dex), salecan (SL), xanthan gum (XG), etc.;
- *algal polysaccharides*: alginic acid (ALG), carrageenan (CG), etc.

Structurally, all polysaccharides consist of long chains comprising monosaccharide units (identical or different) linked by glycosidic bonds. However, they differ by the types of functional groups and their position on the glycosidic skeleton, which dictates their interaction with surrounding matter/molecules.

The available literature on polysaccharide-based composite hydrogels intended for removing pollutants from wastewater is overwhelmingly focused on the synthesis of new matrices and their testing in batch experiments to optimize the sorption conditions (pH, sorbent dose, contact time, equilibrium concentration, temperature) in simulated monocomponent aqueous media [19,21,23,24,25,26,27]. Recently, attention has been directed towards testing the polysaccharide hydrogel-type sorbents in complex matrices of pollutants (competitive investigations) and in column sorption experiments [28,29]. These are pivotal steps towards translating their application to pilot and industrial level set-ups [30]. In this context, here we provide a detailed analysis on the application of polysaccharide-based composite hydrogels as sorbents for pollutant removal from wastewater from a sustainability perspective. Starting with some general considerations of their design principles and of the main analysis strategies of experimental data, the performance of composite hydrogels, classified according to their network architecture, in the sorption of HMIs, organic dyes, and oxyanions from aqueous media is thoroughly evaluated. Emphasis is paid to selectivity and/or reusability data as the main sustainability parameters; works in which composite hydrogels have been tested in column setups or in real wastewater treatment are also presented. Lastly, some general considerations on the main development aspects of composite hydrogels as sustainable solutions for pollutant removal from contaminated waters, as well as the main conclusions and foreseeable development directions, are pointed out.

## 2. Polysaccharide-Based Composite Hydrogels—Design Principles and Adsorption Optimization

**Figure 3:**
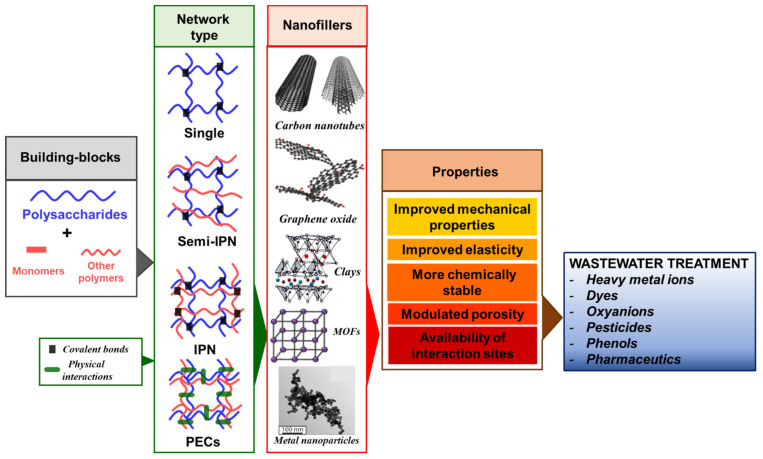
Hydrogel network types and the rational design of different polysaccharide-based composites (containing CNT (Reprinted from Ref. [31]), GO (Reprinted from Ref. [32]), clays (Reprinted with permission from Ref. [33]. Copyright 2019, Royal Society of Chemistry.), MOFs (Reprinted with permission from Ref. [34]. Copyright 2018, John Wiley & Sons, Inc.), or metal nanoparticles (Reprinted with permission from ref. [35]. Copyright 2018, Royal Society of Chemistry.) with improved properties for wastewater treatment.

Hydrogels are cross-linked 3D hierarchical networks composed of synthetic and/or natural polymers that can retain large volumes of water. They can be classified in several ways depending on the: (i) origin of constituent polymers (synthetic, natural, or hybrids); (ii) cross-linking strategy (physical or chemical); (iii) network type (single networks (SN), semi-interpenetrating polymer (semi-IPN) or interpenetrating polymer networks (IPN), and polyelectrolyte complex hydrogels (PECs)) (Figure 3); (iv) charging (non-ionic, cationic, anionic, or ampholytic); or (v) pore mesh size (non-porous, microporous, mesoporous, or macroporous).

Polysaccharide composite hydrogels have been prepared by different strategies, modulating the structure and interactions between constituent components. For example, IPN-type hydrogels are defined according to IUPAC as composite frameworks composed of “*two or more networks, which are at least partially interlaced on a molecular scale, but not covalently bonded to each other and cannot be separated unless chemical bonds are broken*” [36]. Semi-IPN hydrogels are formed when a polymer is evenly dispersed within the cross-linked network of another polymer, without being stabilized by covalent bonds, while IPN hydrogels consist of two distinctly cross-linked intertwined polymeric networks [37]. In case of polysaccharide-based semi-IPN and IPN hydrogels, the first network comprises a polysaccharide, while the secondary component can be either another polysaccharide or a synthetic polymer, thus yielding completely natural or hybrid hydrogels, respectively. The synthetic polymers can be in situ generated within the polysaccharide matrix by polymerizing corresponding monomers [37,38], or can be entrapped and cross-linked (or not) if they are commercially available [39]. IPN-type composite hydrogels with enhanced mechanical strength and toughness have been developed by Gong et al. by the double-network (DN) strategy, mainly for biomedical applications [40,41]. The unique feature of DN hydrogels is the presence of two independent networks, one of which is tightly cross-linked to provide a rigid network, while the other is ductile and loosely cross-linked. DN hydrogels proved to possess not only enhanced mechanical properties but also high performances in environmental applications [42,43,44].

Composite hydrogels, in which the constituent polymers are mutually connected (by physical interactions or chemical bonds), do not fall under the definition of IPN hydrogels. For example, hydrogels stabilized by physical interactions, including electrostatic attractions (defined as polyelectrolyte complex (PEC) hydrogels), hydrogen bonds, hydrophobic interactions, van der Waals forces, or metal coordination [45,46], usually exhibit low mechanical and chemical stabilities, which limits their use in high wear applications. On the other hand, hydrogels prepared by covalent cross-linking are more mechanically stable than the physically cross-linked ones, which is a major advantage for applications such as wastewater treatment. Different covalent cross-linking strategies have been applied to prepare them, including condensation, Schiff base, or click-chemistry reactions, depending on the reactivity of polymer functional groups [39,47,48,49]. However, if small difunctional organic molecules (dialdehydes, dianhydrides, epichlorohydrin (ECH), diglycidyl ethers, etc.) are used as cross-linkers, additional washing steps are mandatory for purification.

Incorporation of nanoscale fillers (Figure 3) within hydrogel networks has been adopted lately as a promising pathway to engineer organic/inorganic composite frameworks with many improved and/or new properties. CNT or GO commonly yield composites with improved flexibility and electronic/ionic conductivity, or respond to near-infrared light [50,51]. Composite hydrogels containing clays show improved elasticity and fast shape self-recovery [19]. Metal nanoparticles (CuNPs, AgNPs, AuNPs, etc.), either introduced in the feed synthetic mixture or synthesized in situ after adsorption of precursor salts, led to materials that exhibit antimicrobial, catalytic, magnetic, plasmonic, or semiconductive properties [52,53]. Lastly, because of their outstanding high surface area, MOFs have also been used lately to fabricate composite hydrogel frameworks for efficient removal of pollutants from water or air [54].

**Figure 4:**
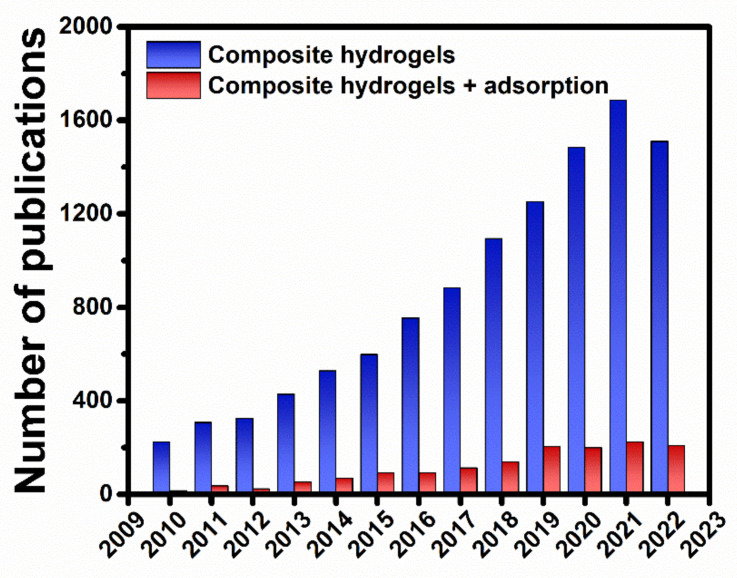
Timeline of the number of publications per year showing the development of the field of composite hydrogels used as sorbents.

Driven by the complementary characteristics provided by polysaccharide chemical structures, potential network architectures, choice of cross-linking method, and nanofillers features, an exhaustive library of composite hydrogels, exhibiting a combination of synergetic properties, has been developed in recent years as potential sorbents for removal of pollutants (HMIs, dyes, oxyanions, pesticides, phenols, pharmaceutics, etc.) from wastewater. The high interest in this field is demonstrated by the timeline presented in Figure 4, showing the yearly number of publications in the last 12 years indexed by the Web of Science™ database. When “*composite hydrogels*” were used as keywords, the number of indexed publications steadily increased year-by-year, up to more than 1500 publication/year. However, when the keyword “*adsorption*” was added as the excluding criterion, it is clearly seen that only a limited number of publications in the “*composite hydrogels*” field refer to their application as sorbents. Nonetheless, more than 200 publications have been published in the last years dealing with “*composite hydrogels*” used for “*adsorption*”.

The sorption performance of composite hydrogels is governed by the presence of a high number of interaction sites on/in the surface/inner pores and the pollutants’ accessibility towards functional groups [55]. Routinely, the sorption experiments are performed in either static (batch) or dynamic (column) setups. Batch experiments provide valuable information on the parameters that influence the composites’ sorption capacity and are used to investigate the pollutants’ sorption mechanisms. Column experiments, however, are intended to emulate the conditions of real industrial wastewater purification installations [28]. Important parameters that affect the sorbent/adsorbate partitioning are the initial pH of the medium, temperature, contact time, sorbent dose, pollutant concentration, bed height, and flow rate (Figure 1C), depending on experiment type. The effect of these parameters will be punctually discussed in the following section.

Nowadays it is customary that the experimental equilibrium and kinetic and dynamic sorption data are fitted with different mathematical models (Table 1) in order to gain information on the interaction between pollutants and sorbents. For example, adsorption isotherms describe the relationship between the pollutant amount retained by the sorbents and the remaining pollutant concentration in solution at equilibrium.

Many isotherm models are available, as reviewed by Al-Ghouti et al. [56], but the most used ones are the two-parameter Langmuir and Freundlich models, and the three-parameter Sips model (equations presented in Table 1). The Langmuir isotherm [57] assumes that all interaction sites have equal affinity towards the adsorbate molecules, the adsorption is homogeneous, and that the thickness of adsorbed layer is one molecule (monolayer) [56,58]. On the contrary, the Freundlich model describes non-ideal adsorption processes, in which a sorbents surface is heterogeneous and multilayer adsorption (due to interactions between the adsorbate molecules) is possible [56]. In the Freundlich isotherm, the parameter *1/n* is related to the surface heterogeneity. Thus, the adsorption is favorable when *1/n* has a value between 0 and 1, it is reversible when *1/n* = 1 and unfavorable when *1/n* > 1. Another important model is the Sips isotherm, a three-parameter model whose expression was developed by combining the Langmuir and Freundlich equations [56]. It follows the Freundlich model at low adsorbate concentration and predicts the monolayer adsorption characteristics by the Langmuir model at high concentrations [59].

Kinetic models also provide important information about the probable mechanism of interaction between pollutants and sorbents. In this regard, the pseudo-first-order (PFO) (Lagergren equation) and pseudo-second-order (PSO) models (Table 1) developed by Ho and McKay [60,61] are the most used models to fit kinetic experimental data. In a PFO model, the adsorption rate is expressed solely as a function of the adsorbate concentration, the rate limiting step being the diffusion process. Hence, it is considered that the adsorption mechanism is controlled by physisorption. On the contrary, the PSO model assumes that the rate limiting step is the electron exchange between adsorbate species and sorbent’s functional groups. In this case, chemisorption is envisaged as the potential mechanism for adsorption.

The kinetic data obtained in column sorption experiments can also be fitted with several mathematical models, among which the Thomas and the Yoon–Nelson models (Table 1) are the most used. The Thomas model has been derived from the Langmuir isotherm model and second-order reaction kinetics assuming the zero longitudinal dispersion of sorbate into the column [62]. The parameter *q_0_* in the Thomas model provides an evaluation of the maximum sorption capacity of sorbents in dynamic conditions. On the other hand, the Yoon–Nelson model assumes that the predicted sorption rate decrease is related to the breakthrough of the adsorbent [63]. In this model, the parameter *τ* gives the time corresponding to 50% breakthrough.

In addition, thermodynamic parameters, such as enthalpy change (**Δ*H°*), Gibbs free energy change (**Δ*G°*), and entropy change (**Δ*S°*) also provide important information on the spontaneity and endo/exothermic nature of sorption processes. **Δ*G°* is determined with Equation (1):

**Δ*G° = −RTln K_c_* (1)

where *K_c_* is the distribution constant, *R* is the universal gas constant (8.314 J/mol∙K), and *T* is the absolute temperature (K). Negative **Δ*G°* values indicate that the sorption of pollutants is favorable and spontaneous. The relationship between **Δ*S°* and **Δ*H°* is given by the Van’t Hoff equation (Equation (2)):

(2) $$lnK_{c}=\frac{\Delta S^{{}^{\circ}}}{R}-\frac{\Delta H^{{}^{\circ}}}{RT}$$

where *K_c_*, *R*, and *T* have the same meaning as above. **Δ*H°* and **Δ*S°* are obtained from the linear representation of *lnK_c_* versus *1/T*. Negative **Δ*H°* indicates an exothermic sorption process, while positive **Δ*H°* corresponds to endothermic sorption. **Δ*S°* is, on the other hand, related to the randomness of solid–liquid interface during pollutant sorption.

Investigation of sorbent selectivity and reusability is, however, of utmost importance from a sustainability perspective. Selectivity quantifies the efficiency of sorbents in retaining target pollutants from complex mixtures. Co-existing species usually reduce or inhibit the sorption of target pollutants, because they compete for the available functional groups, blocking them. Reusability, on the other hand, addresses the sorbents performance in successive sorption/desorption/regeneration cycles. It is usually expressed in terms of regeneration efficiency (*RE*, %), as the ratio between the *n^th^* cycle sorption performance (*q_n_*, mg/g) and the first cycle sorption capacity (*q_1_*, mg/g) (Equation (3)).

(3) $$RE,\;\%=\frac{q_{n}}{q_{1}}\times 100$$

This parameter is related to the working life cycle of materials and is important when aiming to translate the sorbent to pilot scale or industrial level use.

## 3. Polysaccharide-Based Composite Hydrogels Applied in Wastewater Treatment

The following subsections summarize and discuss data related to the performance (maximum sorbed amounts, experimental details, isotherm/kinetic fitting, and selectivity/reusability) of representative composite hydrogels to retain HMIs, dyes, and/or oxyanions, systematized according to their network architecture.

### 3.1. PEC Hydrogels

**Figure 5:**
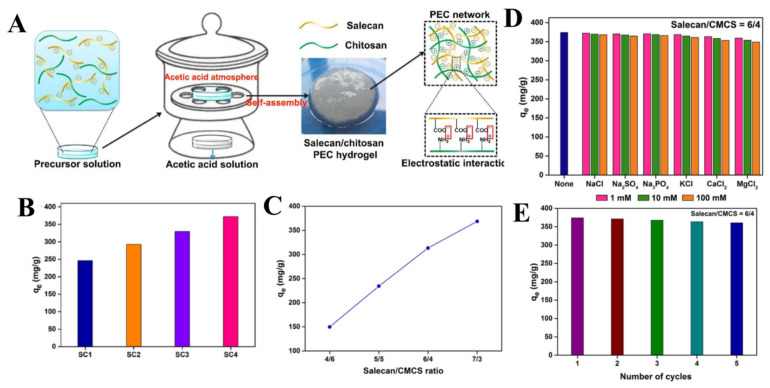
(**A**) The preparation principle of PEC hydrogels by the SD-A-SGT method (the example of SL/CS hydrogels) (Reprinted with permission from Ref. [72]. Copyright 2020, Elsevier.) (**B**) The effect of SL/LCS PEC hydrogels composition (SC1: SL/LCS = 5/5; SC2: SL/LCS = 6/4; SC3: SL/LCS = 7/3; SC4: SL/LCS = 8/2) on Ni(II) sorption (Reprinted with permission from Ref. [73]. Copyright 2020, Elsevier.) (**C**) The effect of SL/CMCS PEC hydrogels composition on Pb(II) sorption (Reprinted with permission from Ref. [74]. Copyright 2020, Elsevier.) (**D**) The effect of competing ions on Pb(II) sorption by the SL/CMCS PEC hydrogels (SL/CMCS = 6/4) (Reprinted with permission from Ref. [74]. Copyright 2020, Elsevier.) (**E**) Reusability efficiency in Pb(II) successive sorption/desorption cycles by the SL/CMCS PEC hydrogels (SL/CMCS = 6/4) (Reprinted with permission from Ref. [74]. Copyright 2020, Elsevier.).

The interest in polysaccharide-based PEC hydrogels has risen lately as a “green” alternative to chemical cross-linked frameworks because of their eco-friendly production without employing toxic reagents and/or solvents [64]. PEC preparation, as nanoparticles, can be tailored by the chemical structure, charge density and concentration of polyelectrolytes, the type and concentration of coexisting small molecular salts, and pH or temperature, as previously demonstrated [65,66,67,68,69]. Since PEC formation is a spontaneous process, the main challenge in shaping PECs into 3D hydrogel architectures (beads or monoliths) and their subsequent stabilization is preventing the occurrence of precipitation [70]. A solution to this problem was recently reported by Zhao et al., who successfully prepared PEC hydrogels based on CS and ALG by a semi-dissolution acidification sol–gel transition (SD-A-SGT) method [71], in which CS was first dispersed in the ALG solution, and then PEC hydrogels were produced by the acidification of the formed suspension using a gaseous acetic acid atmosphere. For example, Figure 5A depicts the preparation principle of PEC hydrogels comprising SL and CS by the SD-A-SGT method [72].

Based on the above method, some polysaccharide-based PEC hydrogels have been recently reported as promising materials for biomedical applications [72], but also for the removal of pollutants from wastewater (Table 2).

For example, Hu et al. prepared PEC hydrogels comprising SL and LCS [73] or CMCS [74] as potential sorbents for Ni(II) ions and Pb(II), respectively. The slow protonation of the amino groups of CMCS or LCS induced the gradual relaxation of polycation chains and their delayed interaction with the carboxyl groups in SL. The sorption performance of thus obtained PEC hydrogels was modulated with respect to SL/polycation ratio, pH, HMI concentration, and contact time. As illustrated in Figure 5B,C, the HMI sorption capacity of the two PEC hydrogels increased as the SL content increased. In addition, pH 6 and pH 7 were found to be the optimal values for Pb(II) and Ni(II) sorption, respectively (Table 2). The experimental sorption capacities for Pb(II) and Ni(II) ions by the SL/CMCS and SL/LCS PEC hydrogels were very high, reaching 415.6 mg/g [74] and 411.8 mg/g [73], respectively. A negligible influence of competing ions in Pb(II) sorption by the SL/CMCS PEC hydrogels (Figure 5D), as well as a remarkable selectivity for Pb(II) when in mixture with Fe(III), Cr(III), Cd(II), Cu(II), Zn(II), Ni(II), Hg(II), and Co(II) ions (Table 2), were noted. The PSO and Langmuir models best fitted the experimental kinetic and equilibrium sorption data, pointing towards a monolayer chemical sorption mechanism. Both types of PEC hydrogels presented more than 95% sorption performance recovery for up to five sorption/desorption cycles (Figure 5E).

In another work, Tang et al. combined the SD-A-SGT method with Ca^2+^-induced internal gelation to prepare CS/ALG DN hydrogels [75]. The obtained physical gels, stabilized by electrostatic interactions between CS and ALG and by Ca^2+^-mediated cross-links of ALG chains, exhibited excellent mechanical properties (maximum tensile strength of up to 0.19 MPa), porous structures, and large specific surface areas, recommending them for wastewater treatment applications. Hence, the composite PEC hydrogels were investigated as sorbents for Pb(II), Cu(II), and Cd(II) ions from monocomponent solutions. Experimental sorption capacities of 176.5 mg/g, 70.83 mg/g, and 81.25 mg/g for Pb(II), Cu(II), and Cd(II) ions, respectively, were determined. Thermodynamic and kinetic studies revealed that the HMI’s sorption was spontaneous, as well as diffusion and reaction controlled. The sorption mechanism, established by X-ray photoelectron spectroscopy (XPS), indicated that Pb(II) and Cd(II) were retained mainly by electrostatic interactions with the −COO^−^ groups, while Cu(II) ions were sorbed by coordination with the amino groups of the composite.

Fast cryostructuration was recently introduced by Dragan et al. to uniformly confine CS powder into aqueous solutions of carboxymethyl cellulose (CMC) (of two molar masses) or poly(2-acrylamido-2-methylpropanesulfonate sodium salt) (PAMPS) (low molar mass), thus yielding pre-PEC sponges [47]. After exposing the obtained pre-PECs to a H^+^ source (such as glacial acetic acid), the protonation of the CS’s amino groups and rearrangement of polyion chains rendered stable and homogeneous PEC sponges. This method has been proven suitable to produce both monoliths and beads with modulated microporosity and exceptional pH-stability, elasticity, and toughness. The prepared CS/CMC PEC sponges also exhibited excellent shape recovery after compression. Despite the slightly laborious preparation pathway, this method offers advantages such as the lack of chemical cross-contaminants and the possibility to engineer materials with different composition, shape, and augmented mechanical properties.

### 3.2. Semi-IPN/IPN Hydrogels

Table 3 summarizes the main compositional and pollutant (HMIs, dyes, oxyanions) sorption aspects of different semi-IPN/IPN composite hydrogels available in the literature.

#### 3.2.1. Semi-IPN/IPN Composite Hydrogels for HMI’s Sorption

Bio-based semi-IPN/IPN hydrogels possess or could be made to bear, by adequate modifications, numerous functional groups (-COOH, -OH, -NH_2_, -SH, etc.) that present effective chelating sites or ion-exchange groups. Among the HMIs, the most scrutinized have been Cu(II) [43,76,77,78,79,80,81,82], Ni(II) [76,78,81], Cd(II) [42,44,76,79,82], Pb(II) [79,80,82,83,84], and Hg(II) [84,85].

pH sensitive semi-IPN Pec/poly(acrylamide-*co*-acrylamidoglycolic acid) hydrogels have been synthesized and tested for the adsorption of Cu(II) and Ni(II) ions, with the maximum adsorption capacities reaching 203.7 mg Cu(II)/g and 121.7 mg Ni(II)/g [78]. The shifts of the main bands in the FTIR spectra of the sorbents loaded with Cu(II) and Ni(II) revealed that these composite hydrogels had a higher affinity for Cu(II) ions than for Ni(II) ions [78]. Semi-IPN hydrogels consisting of XG entrapped in a poly(acrylic acid) (PAA) network have been effective in the adsorption of Cu(II), Ni(II), and Co(II) ions, the order of the adsorption capacity being Cu(II) (530.14 mg/g) > Ni(II) (511.74 mg/g) > Co(II) (436.62 mg/g) [81].

**Figure 6:**
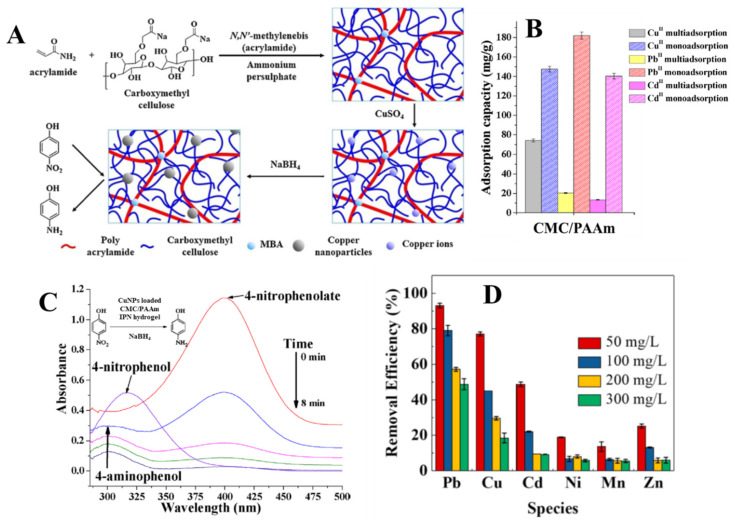
(**A**) The principle of CMC/PAAm semi-IPN hydrogel preparation, sorption of Cu(II) ions, synthesis of CuNPs, and catalytic reduction of 4-NP to 4-AP (Reprinted with permission from Ref. [79]. Copyright 2019 Elsevier.) (**B**) Comparison between Cu(II), Pb(II), and Cd(II) ions sorption by CMC/PAAm semi-IPN hydrogels in mono- and multi-component systems Reprinted with permission from Ref. [79]. Copyright 2019 Elsevier.) (**C**) UV–vis adsorption spectra of 4-NP solution in the presence of CuNPs-loaded CMC/PAAm semi-IPN hydrogel and NaBH_4_ Reprinted with permission from Ref. [79]. Copyright 2019 Elsevier.) (**D**) Selective HMIs removal by lignin/CS/PAAm IPN hydrogel at different initial concentrations (Reprinted with permission from ref. [82]. Copyright 2022 Elsevier).

Zhou et al. developed IPN hydrogels by the functionalization with NH_2_ groups and cross-linking of soluble St, followed by its entrapment in a network of PAA [42]. A sorption capacity of 256.4 mg Cd(II)/g at a sorbent dose of 1 g/L and pH 5.0 was reported in batch experiments for these IPN composites (Table 3). The treated effluent volume of monocomponent Cd(II) solution (200 mg/L) reached 2400 bed volumes after eight sorption/desorption cycles in column experiments. In addition, the adsorption performance loss after one adsorption/desorption/regeneration cycle in dynamic conditions was less than 1 %, which shows that the produced sludge amount is negligible [42]. Composite IPN adsorbents consisting of ALG and GO entrapped in PAA networks, the ALG network being prepared by ionic cross-linking with Ca(II) ions after the formation of PAA network, have been developed by Tang et al. [44]. After freeze-drying, the DN hydrogels displayed a honeycomb morphology with size of pores of about 20–40 µm. Adsorption kinetics of these DN composites for Cd(II) ions was relatively fast, the equilibrium of adsorption being achieved within 120 min at pH 6.0. The sorption kinetics were well fitted by the PSO kinetic model and the sorption at equilibrium was better described by the Langmuir isotherm, with a theoretical maximum sorption capacity of 74.12 mg/g (at 293 K) [44]. Investigation of the selective sorption properties of the designed DN hydrogels showed that the sorption rate increased with the increase in HMI electronegativity, with values of 88.4% for Pb(II) (*χ* = 2.33), 43.4% for Cu(II) (*χ* = 1.9), 33.2% for Cd(II) (*χ* = 1.69), and 12.9% for Mn(II) (*χ* = 1.55). A similar sorption performance order was reported for other composite IPN hydrogels. For example, Ma et al. reported the following order of the maximum sorption capacity for ethylenediaminetetra-acetic acid (EDTA) functionalized CS/poly(acrylamide) (PAAm) IPN hydrogels: 138.41 mg Pb(II), 99.44 mg Cu(II), and 85 mg Cd(II)/g sorbent [43]. In addition, maximum sorption capacities of 312.5 mg Pb(II), 256.4 mg Cd(II), and 227.3 mg Cu(II)/g sorbent (sorbent dose = 0.5 g/L, pH = 5.5) (Table 3) have been reported for semi-IPN CMC/PAAm hydrogels (Figure 6A) in monocomponent HMI solutions [79]. The coexistence of multiple HMIs in common wastewater is known to influence the adsorption efficiency and the adsorption order. Thus, for semi-IPN CMC/PAAm hydrogels, lower q_max_ values were found in a multicomponent Pb(II), Cu(II), and Cd(II) mixture (1:1:1 volumes of 100 mg/L solutions) than in single component HMIs solutions, the adsorption order being Cu(II) > Pb(II) > Cd(II) (Figure 6B) [79]. By in situ reduction of Cu(II) ions, CuNPs-loaded composite hydrogels that could transform 4-nitrophenol (4-NP) in 4-aminophenol (4-AP) have been obtained (Figure 6A,C). This work opens a new avenue in the development of sustainable materials with potential application in circular economy, by the identification of new applications for spent sorbents.

Sorption of Cu(II), Pb(II), and Zn(II) in their mixture with Cd(II) and Ni(II) has been investigated by Zhao et al. on semi-IPN hydrogels comprising CS modified by α-keto-glutaric acid entrapped in a PAAm network, the removal efficiency being 56%, 53% and 38%, respectively [80]. The authors have attributed the preference for Cu(II), Pb(II), and Zn(II) to the electronegativities (*χ*) of these metal ions, which is higher than those of Cd(II) and Ni(II). The radius of hydrated metal ions also has a decisive role in the selectivity of adsorption, the ions with the smallest radius being more easily adsorbed on the hydrogel (Pb(II) has the smallest hydrated radius, its sorption being comparable with that of Cu(II)) [80]. The Langmuir isotherm model well described the adsorption isotherms, with theoretical maximum sorption capacities (at 303 K, 1 g/L sorbent dose and pH 5) for Cu(II), Pb(II), and Zn(II) of 72.39 mg/g, 61.41 mg/g, and 51.89 mg/g, respectively. The sorbent was also highly reusable; more than 90% adsorption capacity recovery for all metal ions after five adsorption/desorption cycles was determined.

Pb(II) ions were successfully removed from aqueous solutions by adsorption on reusable porous semi-IPN hydrogels based on poly(vinyl alcohol) (PVA) entrapped in a matrix consisting of PAA grafted onto ALG [83]. At the optimum PVA content in the composite sorbent (i.e., 2 wt.%) the maximum Pb(II) sorbed amount was 787.4 mg/g sorbent (at sorbent dose = 2 g/L, pH = 5, and 303 K) (Table 3). The semi-IPN hydrogel exhibited a better reusability compared with the control hydrogel without PVA, and about 90% of Pb(II) sorption performance recovery after five sorption/desorption cycles was found [83]. In another work, Ma et al. investigated the removal of Pb(II), Cu(II), and Cd(II) from individual and multicomponent systems by DN hydrogels obtained from lignin extracted from rice husk and PAAm [82]. The developed IPN hydrogels exhibited very fast sorption kinetics (about 10 min), with the estimated theoretical maximum Pb(II), Cu(II), and Cd(II) sorbed amounts being 374.32 mg/g, 196.68 mg/g, and 268.98 mg/g, respectively (Table 3). The relative selectivity coefficients in multicomponent systems suggested that this composite IPN hydrogel adsorbed Pb(II) preferentially, followed by Cu(II) and Cd(II) (Figure 6D) [82].

#### 3.2.2. Semi-IPN/IPN Composite Hydrogels for Dye Sorption

Many semi-IPN/IPN composite hydrogels having at least one polysaccharide in their composition have also been evaluated for their performance in the removal of cationic [86,87,88,89,90,96,97,98,99,100,101,102,103,104,105] and anionic [91,92,97,103,104,105] dyes in single- or multi-component [93] solutions. Since most dyes contain ionic functional groups in their structure, the sorption mechanism is mainly driven by the electrostatic interactions between them and the functional groups of the sorbents, with hydrogen bonds also having a contribution [98,100]. The concentrations of H^+^ and OH^−^ ions also play a decisive role in the adsorption process since they can lead to protonation or deprotonation of functional groups on the adsorbent surface [91,98,100].

IPN hydrogels, prepared by free radical cross-linking polymerization of acrylamide (AAm) in the presence of CS [86,97] under freezing conditions followed by CS cross-linking with ECH under alkaline conditions, have been tested by our group for the sorption of MB in batch experiments. During the CS cross-linking step, the partial hydrolysis of amide groups in the PAAm network to carboxylate groups was simultaneously achieved. The developed IPN sorbents were capable of retaining 750 mg MB/g composite (sorbent dose 1 g/L, pH 5.5, 298 K) (Table 3) and even of selectively recovering MB from its mixture with MO [86]. Semi-IPN cryogels, consisting of anionically modified potato St entrapped in a PAAm matrix by a similar strategy [87], were capable of retaining 443.7 mg/g of MB, which was further increased up to 667.7 mg/g by the alkaline hydrolysis of PAAm. The MB sorption at equilibrium was well described by the Sips isotherm model, while the sorption kinetics was well described by the PSO model, supporting chemisorption as the main removal mechanism [87]. Notably, the MB sorption capacity remained almost unchanged after six successive sorption/desorption cycles and the composite sorbent preserved its integrity, which proved that the prepared IPN sorbent could be sustainably used in removing dyes from industrial effluents.

In another work, IPN composite hydrogels prepared by free radical cross-linking polymerization of acrylic acid (AA) in the presence of PVA and yeast cells have been investigated by Feng et al. for the sorption of MB [90]. A maximum MB sorbed amount of 629 mg/g (sorbent dose 1 g/L, pH 8, and 303 K) was reported for these developed hydrogels (Table 3). Contrasting affinity and selectivity between cationic and anionic dyes were ascertained at different pH values as a result of the pH-dependent protonation/deprotonation of hydrogel functional groups.

#### 3.2.3. Semi-IPN/IPN Composite Hydrogels for Oxyanions Sorption

Among oxyanions, phosphate removal from wastewater is receiving extensive attention nowadays because concentrations exceeding 1 ppm are responsible for the eutrophication process, which causes a dramatic decline in the oxygen dissolved in water and deterioration of the ecological equilibrium. To this end, several semi-IPN/IPN composite hydrogels have been recently developed and inspected for their phosphate removal performance from wastewater [39,94,95].

**Figure 7:**
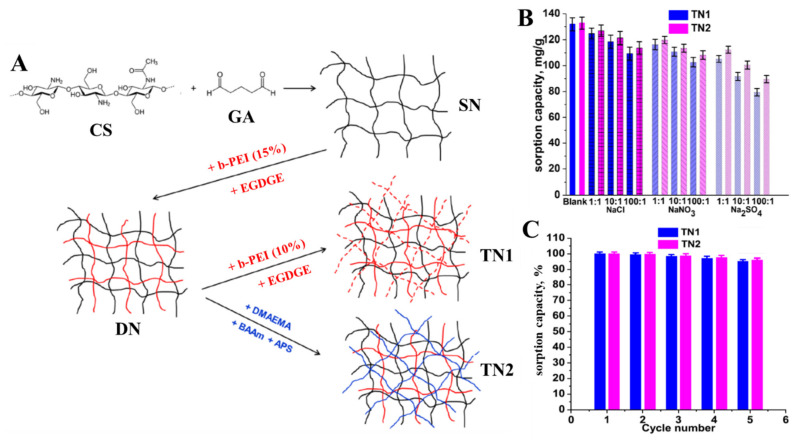
(**A**) The preparation strategy of CS/PEI/PEI and CS/PEI/PDMAEMA TN sponges (Adapted with permission from ref. [39]. Copyright 2021 Elsevier.) (**B**) The influence of interfering anions on the phosphate sorption by CS/PEI/PEI and CS/PEI/PDMAEMA TN sponges (Reprinted with permission from ref. [39]. Copyright 2021 Elsevier.) (**C**) Phosphate sorption/desorption cycles CS/PEI/PEI and CS/PEI/PDMAEMA TN sponges (Reprinted with permission from ref. [39]. Copyright 2021 Elsevier.).

For example, Wan et al. have developed composite sorbents as beads, with zirconium loaded into magnetic CS/PVA IPN hydrogels for phosphate recovery [94]. The maximum phosphate sorbed amount at pH 5 was 50.76 mg/g sorbent, and the composite IPN hydrogel beads were also successfully reused for up to five cycles without any loss of the initial sorption capacity. DN sponges comprising CS, as a polycation from renewable resources, and polyethyleneimine (PEI) or poly [2-(dimethylamino)ethyl methacrylate] (PDMAEMA) as synthetic polycations have been prepared using the ice-templating strategy and evaluated by Dragan et al. for their performance in the removal of phosphate oxyanions from aqueous media [95]. To further increase the phosphate sorption capacity of the biocomposite sponges, and also to improve their mechanical strength, a third network (TN) of synthetic polycation (PEI or PDMAEMA) was engineered inside the pores of DN sponges [39]. The synthesis pathway for single network (SN), DN, and TN sponges is schematically presented in Figure 7A.

The inner morphology of the as-prepared composite sponges changed with each modification step, varying from an open pore aspect for the SN hydrogel to a clogged pore-look in the case of DN and TN composites. It was ascertained that phosphate desorption was stimulated by the presence of PDMAEMA in the TN sponges and by increasing the temperature (up to 35 °C). The influence of interfering anions on phosphate removal by TN sponges followed the order SO_4_^2−^ > NO_3_^−^ > Cl^−^, and increased as their concentration increased (Figure 7B). The sustainability of TN sponges in phosphate removal was further demonstrated by the 95% sorption performance recovery after five successive sorption/desorption cycles (Figure 7C), desorption being performed by using 1 M NaOH as eluent. To augment the sponges’ selectivity for H_2_PO_4_^−^ ions, hydrated iron oxide (HFO) nanoparticles were also generated in situ into the DN and TN cryogels [39]. The mechanism of phosphate sorption by the HFO-doped TN sponges involved an interplay of electrostatic attractions and inner sphere complexation.

### 3.3. Other Polysaccharide Composite Hydrogels

Other polysaccharide composite hydrogels, different from PECs and semi-IPN/IPNs, were also reported in recent years as versatile and sustainable solutions to remove pollutants from wastewater. The interest in such materials originates from their multiple benefits such as straightforward preparation pathways, low production costs, robust mechanical features, high number of functional groups, and adaptability to column setups. Some recent examples of such composites and their performance in the sorption of HMIs, dyes, and oxyanions are presented in Table 4 and discussed in the following sub-sections.

#### 3.3.1. Other Polysaccharide Composite Hydrogels for HMI Sorption

**Figure 8:**
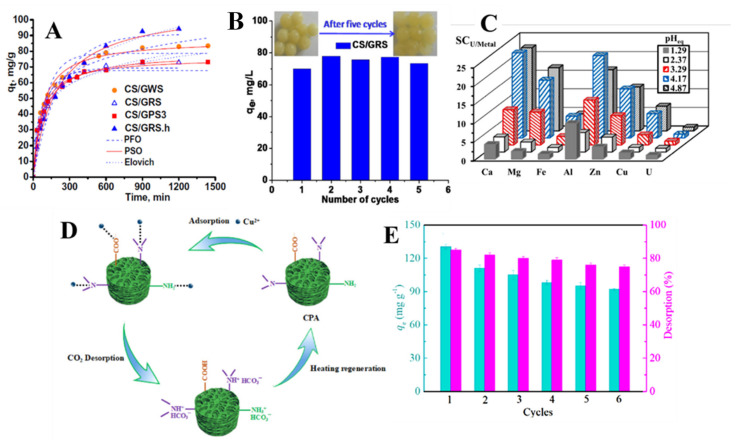
(**A**) The effect of contact time on Cu(II) sorbed amount by CS/PAN-*g*-St composite beads differing by St source and hydrolysis (Reprinted with permission from Ref. [106]. Copyright 2022 Elsevier.) (**B**) Cu(II) removal performance by CS/PAN-*g*-St (from rice) composite cryobeads in successive sorption/desorption cycles (Reprinted with permission from Ref. [106]. Copyright 2022 Elsevier. (**C**) Effect of pH on selective removal of U(VI) by the sulfonate modified CS/Arabic gum biosorbent in the presence of competitive HMIs (Reprinted with permission from Ref. [107]. Copyright 2022 Elsevier.) (**D**) Illustration with the cyclic Cu(II) sorption, CO_2_-mediated desorption, and regeneration of P(AA-*co*-DMAEMA)/CS aerogels (Reprinted with permission from ref. [108]). (**E**) Cu(II) sorption/CO_2_-mediated desorption performance of P(AA-*co*-DMAEMA)/CS aerogels (Reprinted with permission from ref. [108]).

In our group, composite biosorbents were prepared as beads, using CS and polyacrylonitrile (PAN)-*g*-St [106] or poly(amidoxime) (PAMOX)-*g*-St [23,126] by dual cross-linking methodologies for HMI removal from simulated wastewater. For example, CS/PAN-*g*-St beads obtained by dual chemical cross-linking using GA and poly(ethylene glycol diglycidyl ether) through a fast cryostructuration methodology (dropping the precursor mixture composed of grafted St powder, CS solution, and cross-linkers into liquid nitrogen) were investigated as sorbents for Cu(II), Ni(II), and Co(II) ions [126]. Among different St sources (potato, wheat, and rice, abbreviated with GPS.3, GWS, and GRS, respectively, in Figure 8A,B), the composites prepared using rice St gave the highest HMI sorption performance (Figure 8A), probably due to a more favorable amylose to amilopectin ratio [127]. It was also showed that the HMI sorption capacity could be further augmented by the hydrolysis of nitrile groups in PAN. Experimental sorption capacities for Cu(II), Ni(II), and Co(II) of 100.6 mg/g, 83.25 mg/g, and 74.01 mg/g, respectively, were determined for CS/PAN-*g*-rice St composite hydrogels (at pH 5 for Cu(II) and pH 6 for Ni(II) and Co(II), 300 K, and 1 g/L sorbent dose). The isotherm and kinetic results were best fitted by the Langmuir and PSO models, indicating a monolayer chemisorption mechanism. The sorbents’ sustainability was supported by their complete sorption capacity recovery and structural integrity preservation after five sorption/desorption cycles (Figure 8B) [106].

Glucan/CS hydrogels prepared by Jiang et al. by ultrasound-assisted free radical cross-linking polymerization have demonstrated remarkable sorption capacity towards multiple HMIs [27]. Thus, maximum sorbed amounts of 342 mg Cu(II), 232 mg Co(II), 184 mg Ni(II), 395 mg Pb(II), and 269 mg Cd(II) per gram of hydrogel were recorded (at pH 7, 293 K, and 3h contact time) for the above-prepared hydrogel. The thermodynamic, isotherm, and kinetic investigations, as well as the fitting results, revealed that the adsorption was a spontaneous monolayer chemisorption process.

Bio-based composite hydrogels comprising CS and arabic gum recently developed by Hamza et al. also showed a high versatility in removing multiple HMIs from simulated and real wastewater [107]. By functionalization of the composites with 2-[(p-aminophenyl)sulfonyl]ethyl hydrogen sulfate and 2-acrylamido-2-methyl propane sulfonic acid (AMPS), a maximum U(VI) sorbed amount of 471.24 mg/g was achieved at a contact time of about 20 min (at pH 4 and 325 K). The developed composites were highly sustainable; their sorption performance recovery after five sorption/desorption cycles was higher than 97%. At pH > 4.17, the composite sorbents were highly selective for U(VI) ions compared to Ca(II), Mg(II), Al(III), and Zn(II) ions, but lower against Cu(II) and Fe(III) (Figure 8C). A remarkable removal performance of metal ions (U(VI), Cu(II), Fe(III), Zn(II), and Pb(II)) from real water samples collected from five wells in Sinai (Egypt) was also demonstrated. Corroborated with a negligible toxicity (against normal and cancerous cell lines) and good antimicrobial performance against multiple bacterial pathogens, the sorbent could fulfill the strict requirements of providing safe drinking water in mining areas.

Concerns have lately been raised over currently used HMI desorption strategies (acidic solutions of chelating agents) from spent sorbents that produce large volumes of concentrated secondary pollutants, whose neutralization greatly increases the environmental impact and total operation cost of adsorption setups [128]. To circumvent this drawback, an alternative strategy was recently proposed by Fan et al., who exploited the CO_2_-responsivity of P(AA-*co*-DMAEMA)/CS aerogels to promote the desorption of retained Cu(II) ions [108]. The prepared P(AA-*co*-DMAEMA)/CS aerogels reached a maximum sorption capacity for Cu(II) ions of 131.6 mg/g at a contact time of 8 h, pH 6, and 298 K (Table 4). Cu(II) desorption was induced by CO_2_ bubbling into the release medium (distilled water) for up to 6 h. CO_2_ significantly decreased the medium’s pH (down to pH 4.2), thus protonating the -NH_2_ and -N< groups within the hydrogels and promoting the metal ion desorption by charge repulsions (Figure 8D). Applying this desorption method, Cu(II) sorption capacity after 6 cycles was still higher than 70% of the initial value, while the desorption rate reached 75% (Figure 8E). As a resul of the CO_2_-response features of P(AA-*co*-DMAEMA) within the aerogel, the successive sorption/desorption cycles did not produce any side-products [108].

**Figure 9:**
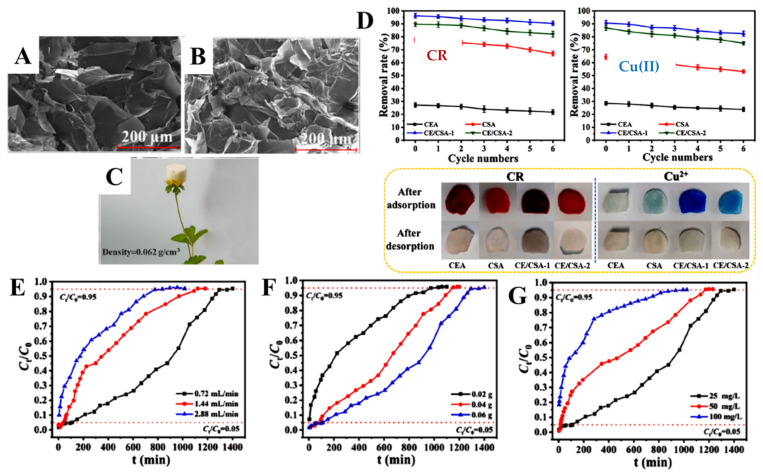
SEM micrographs of CEL/CS aerogels prepared at 1 wt.% (**A**) and 2 wt.% (**B**) CS solution. (**C**) Optical image of CEL/CS aerogel prepared at 1 wt.% CS solution featuring its ultralightweight property. (**D**) Reusability performance and optical images after pollutants sorption and desorption of CEL/CS aerogel (abbreviated with CE/CSA-1 and CE/CSA-2, the number identifying the concentration of CS solution), as well as of control CEL (CEA) and CS (CSA) aerogels. The breakthrough curves of CR sorption in column experiments by the CEL/CS aerogel prepared at 1 wt.% CS solution as a function of (**E**) flow rate, (**F**) sorbent mass and (**G**) pollutant concentration. (Reprinted with permission from Ref. [109]. Copyright 2023 Elsevier.)

Green CEL/CS aerogels with a hierarchical 3D porous architecture and low density (0.062 g/cm^3^) were recently prepared by Liu et al. by combining sol–gel and freeze-drying methods using ECH as a cross-linker [109]. Figure 9A,B shows the SEM micrographs of aerogels prepared at 1 wt.% and 2 wt.% CS solution, respectively, showing their macroporous interconnected inner pores. The aerogels also had a very low weight, easily supported even by a flower (Figure 9C).

The aerogels were capable of retaining up to 255.1 mg/g of CR and 202.43 mg/g of Cu(II) ions from single-component simulated wastewater. However, the sorption capacity of CEL/CS aerogels for CR and Cu(II) was augmented by about 49% and 28.6%, respectively, when the two pollutants were mixed together, the maximum of their sorption capacity thus reaching 380.23 mg/g and 260.41 mg/g, respectively. This was assigned to a synergetic influence of the two pollutants. Thus, preadsorbed CR in the aerogels brings extra -NH_2_ and -SO_3_^−^ groups that can interact with Cu(II) ions by chelation and electrostatic attractions. At the same time, preadsorbed Cu(II) ions provide extra interaction sites to promote the adsorption of CR by the CEL/CS aerogels. The aerogels also exhibited a high reusability performance (>90%) with albeit a slightly cumbersome desorption/regeneration procedure required to preserve their porous structure (Figure 9D). Moreover, the CEL/CS aerogels retained up to 241 mg CR/g sorbent in fixed-bed column experiments (Figure 9E–G), which strengthens their potential for practical wastewater treatment applications.

#### 3.3.2. Other Polysaccharide Composite Hydrogels for Dyes Sorption

A straightforward method to prepare organic composite hydrogels for dyes sorption is by grafting polymerization of acrylic monomers (AA, AAm, AMPS, sodium allylsulfonate, etc.) onto polysaccharide backbones [113,117,118,119,120,129,130]. The preparation of such composites usually consists of the following two steps: (i) the preparation of a solution containing the polysaccharide (CS, XG, ALG, CEL) and desired amounts of acrylic monomers and cross-linkers (such as *N*,*N*’-methylenebisacrylamide (BAAm)); (ii) the addition of the polymerization initiator (for example ammonium persulfate) into the solution and assuring conditions for its dissociation into radicals. By this approach, Gohari et al. recently prepared XG-g-P(AAm-co-AMPS) hydrogels that have been tested as sorbents for MB [119]. The optimization of the hydrogel’s composition for MB removal was performed with respect to AMPS, AAm, and MBA composition. It was found that the best MB removal capacity was obtained when the feed concentration of AAm, AMPS, and MBA were 6 wt.%, 4.09 wt.%, and 0.12 wt.%, respectively. The maximum sorption capacity was 384.62 mg MB/g, the sorption data being best fitted by the Langmuir isotherm and PSO kinetic models. In addition, a good reusability of 83.5% after six sorption/desorption cycles was reported for this composite hydrogel.

To improve the dyes adsorption capacity of hydrogels, it is mandatory to increase the number of functional groups in their structure. In this respect, Chen et al. recently designed a new MB superadsorbent composite hydrogel by cross-linking grafting polymerization of AA onto St [129]. A sorption capacity of 2967.66 mg/g for MB was reported for the optimum hydrogel that had the highest grafting parameters: 90.79% grafting efficiency, 50.06% reaction ratio at C_6_ in St, and 248.49% grafting ratio of PAA [129]. The MB adsorption was an endothermic and spontaneous process and obeyed the Langmuir and PFO fitting models. After five sorption/desorption steps, the hydrogel still was capable of retaining 2137.5 mg MB/g, which corresponds to a level of reusability of about 72%.

Besides the abundance of functional groups, their type (anionic, cationic, or neutral) is important in tuning the affinity of hydrogels toward specific dyes, which could be useful in dye separation from complex wastewater effluents. To address this aspect, Ilgin et al. prepared a series of composite hydrogels by cross-linking polymerization of AA, AAm, or 3-(acrylamidopropyl)trimethyl ammonium chloride (APTMACl) in the presence of hydroxyethyl-St (HESt) [117]. Hence, the obtained hydrogels contained anionic, neutral, and cationic moieties, respectively. The hydrogels sorption performance towards MO (anionic dye) and MV (cationic dye) largely depended on the hydrogel composition. Thus, the cationic hydrogel HESt/P(APTMACl) sorbed preferentially MO (238.1 mg/g) but had a very low sorption capacity for MV (6.62 mg/g). On the contrary, the anionic HESt/PAA sorbed 185.2 mg MV/g, but only 2.84 mg MO/g. Finally, the HESt/PAAm hydrogels, which were neutral, had low sorption capacities for both dyes (Table 4). Hence, Coulomb attractions are the main interactions involved in dye sorption, with minor contributions from hydrogen bonds or van der Waals forces. The isotherm and kinetic experimental data were well fitted by the Langmuir and PFO models [117], thus indicating a monolayer physisorption mechanism.

#### 3.3.3. Other Polysaccharide Organic Composite Hydrogels for Oxyanion Sorption

Different polysaccharide-based organic composite hydrogels have also been developed lately for the removal of oxyanions from wastewater [121,122,123,124,125,131,132]. Cr(VI), commonly found as chromate (Cr_2_O_4_^2−^) or dichromate (Cr_2_O_7_^2−^), is an extremely dangerous pollutant, being considered an important human carcinogen. A highly potent composite biosorbent for Cr(VI) ions was recently prepared by Huang et al., as beads, from sodium lignosulfonate (NaLS), PEI, and ALG by a combination of chemical and ionotropic cross-linking [125]. The composite exhibited a maximum Cr(VI) sorption capacity of 2500 mg/g at pH 2 and 298 K, with the sorption equilibrium being achieved in about 6 h (Table 4). A minor influence of competing pollutants (humic acid, Cl^−^, NO_3_^−^, and SO_4_^2−^) was noted, demonstrating the high affinity of the sorbent for Cr(VI). In column studies, 1 g of NaLS/PEI/ALG composite was capable of purifying up to 8.1 L of industrial secondary electroplating wastewater, with the Cr(VI) concentration in the effluent meeting the discharge standard regulated concentration (<0.2 mg/L) [125].

**Figure 10:**
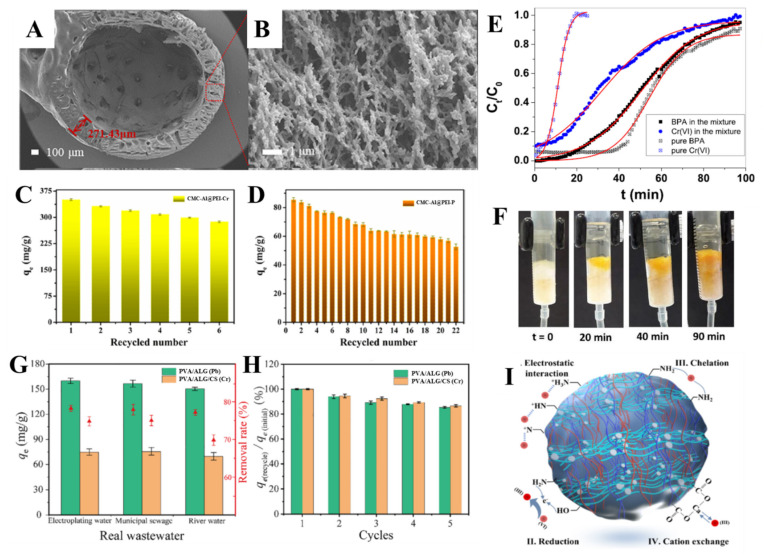
SEM micrographs of hollow CMC/PEI beads (**A** and **B**) and their reusability performance in the removal of Cr(VI) (**C**) and phosphate (**D**) (Reprinted with permission from Ref. [122]. Copyright 2021 Elsevier.) (**E**) Breakthrough curves (with Thomas model fitting profiles) for bisphenol A and Cr(VI) sorption onto CTAB-modified CMC/sugarcane baggase composite in monocomponent solution and in mixture, and (**F**) photographs at different time intervals of the column during bisphenol A and Cr(VI) sorption (Reprinted with permission from Ref. [131]. Copyright 2022 Elsevier.) Pb(II) and Cr(VI) ions sorption by PVA/ALG and PVA/ALG/CS composite hydrogels, respectively, from real wastewater (**G**), their reusability performance (**H**) and illustration of the possible interactions between Cr(VI) ions and PVA/ALG/CS hydrogels (**I**) (Reprinted with permission from Ref. [111]. Copyright 2022 Elsevier.)

Hollow composite beads for the sorption of both Cr(VI) and phosphate were prepared by Yang et al. through the surface modification of CMC beads with PEI and subsequent GA cross-linking [122]. The beads’ hollow structure (Figure 10A) and porous shell (with about 271.43 µm thickness) (Figure 10B) endow the composite with numerous accessible sorption sites. These features promoted a high adsorption capacity for Cr(VI) and phosphate (535.39 mg/g and 150.65 mg/g, respectively) and fast adsorption kinetics (400 min and 200 min, respectively) up to equilibrium.

The optimum sorption pH was 2 for Cr(VI) and 3 for phosphate, as also reported in other works [95,124,125]. The beads recyclability was excellent, only a low sorption performance decline for Cr(VI) and phosphate after 6 (Figure 10C) and 22 (Figure 10D) successive sorption/desorption cycles, respectively, was determined. The sorption of the two oxyanions occurred mainly by electrostatic interactions (as demonstrated by XPS, FTIR, and zeta potential measurements), but for Cr(VI) a contribution from its reduction to Cr(III) was also noted [122]. Phosphate adsorption in column experiments was also excellent; the maximum sorption capacity predicted by Thomas model was 167.55 mg/g and 205.02 mg/g at 1 mL/min and 3 mL/min flow rates, respectively.

The presence of cross-contaminants in real wastewater effluents could have a promoting or an inhibitory effect on oxyanions sorption, as well as for other pollutants. In this regard, Meneses et al. recently investigated the effect of coexisting contaminants (MB and bisphenol A) on Cr(VI) sorption in column setups by cryogels composed of CMC and sugarcane baggase, that were also doped with cetyltrimethylammonium bromide (CTAB) micelles [131]. A 3.5-times enhancement in the Cr(VI) sorbed amount was found in its binary (with bisphenol A) and ternary (with MB and bisphenol A) mixtures compared with the single component solution, while a 1.4-times increase of Cr(VI) sorption capacity was determined when it was mixed with MB. The breakthrough curves obtained in the binary Cr(VI) and bisphenol A system also highlighted the synergetic sorption of the two pollutants (Figure 10E). The Thomas model predicted a maximum Cr(VI) sorbed amount increase from 3.07 mg/g in single component system to 10.33 mg/g in the binary system. Photographs taken at different column running times (Figure 10F) clearly show the advancement of mass transfer zone, until exhaustion was reached after 90 min. This composite was also successfully used in five sorption/desorption cycles without significant performance decline, demonstrating its very good regenerability.

PVA/ALG and PVA/ALG/CS composite hydrogels as beads, recently prepared by Zhang et al. by cross-linking blending, have been successfully used to remove both HMIs and oxyanions from wastewater [111]. Maximum Pb(II) and Cr(VI) sorbed amounts of 139.4 mg/g and 86.1 mg/g have been reported for the PVA/ALG and PVA/ALG/CS hydrogels, respectively. The presence of CS in the ternary composite hydrogel significantly improved its selectivity for Cr(VI). The sorption of Pb(II) and Cr(VI) was endothermic, and was dominated by chemisorption and physisorption, respectively. The two composite hydrogels demonstrated very good Pb(II) and Cr(VI) sorption properties from real wastewater (electroplating, municipal, and river) (Figure 10G), the presence of cross-contaminants even improved the sorption of Pb(II) ions. After five sorption/desorption cycles (Figure 10H), the Pb(II) and Cr(VI) removal performance of the hydrogels was still higher than 85 % compared to initially. The sorption mechanism, established by FTIR, FESEM-EDS, and XPS, indicated Cr(VI) ions were retained by an interplay of electrostatic interaction, reduction, chelation, and cation exchange (Figure 10I) [111].

### 3.4. Organic/Inorganic Composites

Lately, a strong interest has also been addressed to the development of novel and more efficient composite biosorbents based on polysaccharides and inorganic fillers (clays, zeolites, GO, or magnetic nanoparticles) and on their application in the treatment of wastewater (Table 5). This growing attention on organic/inorganic composite sorbents is associated with their advantages in the separation processes, such as their improved sorption capacity, fast kinetics, and remarkable mechanical and chemical stability during regeneration and reusability studies [19,133,134,135,136].

#### 3.4.1. HMI Sorption by Organic/Inorganic Composites

CS coated montmorillonite (MMT) beads cross-linked with ethylene glycol diglycidyl ether have been prepared and tested for removal of Cu(II), Ni(II), Pb(II), and Zn(II) ions from their mono- and multi-component aqueous mixtures [137]. The equilibrium of sorption was attained in 1 h at pH 8, while the maximum sorption capacity in mono- and multi-component systems followed the order: Pb(II) > Zn(II) > Cu(II) > Ni(II) (Table 5). Moreover, the feasibility of these adsorbents for removal of Cu(II), Ni(II), Pb(II), and Zn(II) ions from groundwater was also investigated. A removal efficiency of 94.08%, 92.42%, 88.28%, and 42.04% was found for Pb(II), Cu(II), Ni(II), and Zn(II) ions, respectively.

**Figure 11:**
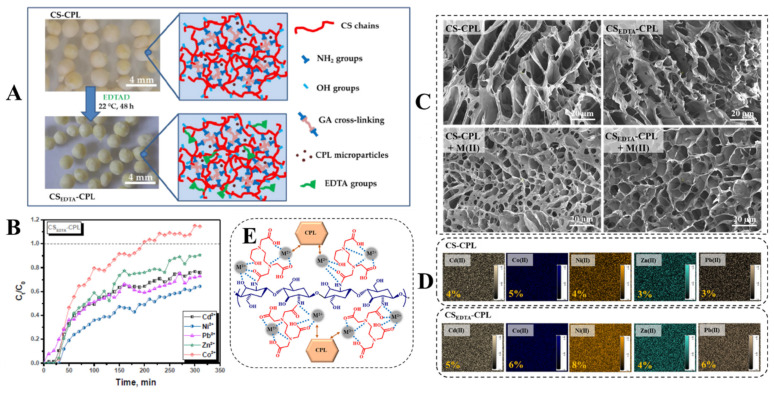
(**A**) Optical images of CS-CPL and CS_EDTA_-CPL beads and illustration of functional groups (-NH_2_, -OH, and EDTA) available to interact with HMIs. (**B**) Breakthrough curve of Cd(II), Ni(II), Pb(II), Zn(II), and Co(II) ions sorption (from their mixture) on CS_EDTA_-CPL beads. (**C**) SEM micrographs of CS-CPL and CSEDTA-CPL beads before and after interaction with a equimolar mixture of Cd(II), Ni(II), Pb(II), Zn(II), and Co(II) ions. (**D**) EDX mapping and elemental analysis of the CS-CPL and CS_EDTA_-CPL bead surface after interaction with a mixture containing Cd(II), Ni(II), Pb(II), Zn(II), and Co(II) ions. (**E**) Potential interaction mechanism between the CS_EDTA_-CPL composite cryobeads and HMIs (Reprinted from Ref. [29]).

An original approach which combines ion-imprinting and cryogelation techniques has been developed by Dinu et al. [169,170] to prepare CS-based sorbents with high affinity and selectivity for Cu(II) ions. Thus, advanced sorbents comprising CS, CPL, and PAAm were prepared by in situ synchronous cross-linking of pre-formed Cu(II)/CS complexes by GA and polymerization of AAm in the presence of BAAm and a natural zeolite (CPL). These sorbents were successfully used in selective removal of Cu(II) ions from its binary or multicomponent mixtures with Co(II), Ni(II), Zn(II), and/or Pb(II) ions [169]. In addition, the sorption equilibrium was rapidly reached (20 min) as well as very fast complete elution of the HMIs adsorbed [170]. This behavior was associated with the existence of pre-organized recognition sites along with a highly interconnected porous morphology within the sorbents structure. Furthermore, as the industrial wastewater comprise mixtures of a broader variety of heavy metal ions, our group also evaluated the adsorption capacity of CS/CPL composite sorbents for Cu(II) ions from ternary or five-component synthetic mixtures with Zn(II), Ni(II), Fe(III), and Cr(III) ions [171,172,173]. It was shown that the removal efficiency of HMIs from their aqueous mixtures strongly depended on several characteristic parameters of the aqueous media, such as the presence of other contaminants or competing species, ionic strength, temperature, and pH [171,172,173]. The overall sorption tendency of CS/CPL sorbents toward Cu(II) ions in the presence of Zn(II), Ni(II), Fe(III), and Cr(III) ions, under competitive conditions, followed the order: Cu(II) > Fe(III) > Ni(II) > Zn(II) > Cr(III) with distribution coefficients values of 4.23, 2.74, 1.43, and 55.9. The efficiency of ion-imprinted CS/CPL sorbents was also demonstrated in real-life aqueous effluents discharged from photo-etching processes [171]. A removal efficiency of 98.89%, 94.56%, 91.67%, 92.24%, and 82.76% was achieved for Cu(II), Fe(III), Ni(II), Zn(II), and Cr(III) ions, respectively, using a sorbent dose of 6 g/L. To further increase the chelation performance of these sorbents, the functionalization with aminopolycarboxylic acids such as EDTA and diethylenetriaminepentaacetic acid (DTPA) has been considered [29,173]. The investigation of Cu(II), Co(II), and Fe(III) ions sorption in binary and ternary systems indicated that all composite sorbents exhibited a high affinity for Fe(III) ions. The experimental sorption capacity values for removal of Fe(III) ions from their mixture with Cu(II) ions by CS/CPL, EDTA-modified CS/CPL, and DTPA-modified CS/CPL sorbents were 161.60 mg/g, 189.61 mg/g, and 206.65 mg/g, respectively. Moreover, these composite sorbents displayed a remarkable chemical stability during desorption/regeneration processes; their sorption capacity remained almost unchanged even after the fifth cycle of sorption [173]. To check their industrial feasibility, sorption studies were further performed in fixed-bed column set-ups using five-component HMI solutions containing Zn(II), Pb(II), Cd(II), Ni(II), and Co(II) in equimolar concentrations [29]. In this regard, chemically cross-linked CS/CPL composite sorbents as beads were prepared as beads by a cryogelation process and were further reacted with 4,4′–ethylenebis(2,6–morpholinedione) (EDTAD) to introduce EDTA ligand moieties in the matrix (Figure 11A).

A maximum theoretical HMI sorption capacity of 145.55 mg/g and a 50% breakthrough time of 121.5 min were calculated for the columns containing CS_EDTA_-CPL sorbents using Thomas and Yoon–Nelson models. The fixed-bed column sorption experiments indicated the following affinity trend for the CS_EDTA_-CPL sorbent: Co(II) < Zn(II) < Cd(II) < Pb(II) < Ni(II), showing that Co(II) ions exhibit the lowest affinity for the sorbent functional groups, whereas Ni(II) ions were strongly bound (Figure 11B). The drastic decrease in pore sizes after M(II) sorption (Figure 11C) and the presence of the Ni(II) ions in the highest ratio on the sorbent surface (Figure 11D) demonstrated the strong interaction between HMIs and functional groups of the support matrix. The possible mechanism of HMI sorption by CS_EDTA_-CPL sorbent is shown in Figure 11E. Overall, it was demonstrated that the functionalization of CS-based composites with APCA moieties is a powerful strategy to significantly enhance their HMIs chelation performances.

In another work, Ahmad et al. prepared zeolite-containing XG-glutathione biocomposites by the melt intercalation technique and investigated their efficiency in removal of both HMIs (Ni(II) and Pb(II)) and anionic dyes (i.e., CR) [141]. The equilibrium of sorption was attained after 2 h for Ni (II) and Pb (II) ions and after 4 h for CR. The optimum pH for Ni(II), Pb(II), and CR removal was established as 4, 5, and 2.1, respectively (Table 5). Even if these biocomposites exhibited good sorption performance for both HMIs and anionic dyes, the decrease in the pollutant desorption to about 60% after five cycles of regeneration limits their feasibility for industrial application. Environmental friendly composite biosorbents have also been developed by Filla et al. [140] by modifying ALG with biochar and CPL, and tested for adsorption of rare earth elements, i.e., La(III), Ce(III), Pr(III), and Nd(III) ions. The ALG/biochar sorbents exhibited much higher sorption capacities than the CaALG/CPL composites (Table 5). The La(III), Ce(III), Pr(III), and Nd(III) ions sorption by ALG/biochar and ALG/CPL was significantly affected by the initial pH of the aqueous solution, sorbent dose, contact time, initial metal ion concentration, temperature, and the presence of competing ions. HNO_3_ was the best desorbing agent, and the sorbent composites can be reused in up to six adsorption/desorption cycles with removal efficiencies of over 97% [140].

To enhance the mechanical and chemical stability of various polysaccharides the embedding of GO [142,143,164] or metal oxides [144,146,147,148,149,164] such as Fe_3_O_4_ or MnO_2_ has also been considered. For example, Hu et al. developed SL/GO sponges by combining ice template-assisted freeze-drying and ion-imprinting technologies with high potential for removal of Hg(II) [142] and Cd(II) ions [143]. These materials exhibited a macroporous structure with 3D interconnected channels, which allowed the easy and fast diffusion of Hg(II) ions to the sorbent binding sites. The Hg(II) sorption capacity depended on the SL content, pH, initial HMI concentration, pore size distribution, and contact time. A maximum sorption capacity of 413.6 mg Hg(II)/g sorbent was achieved at pH 7 in 1 h [142]. Outstanding sorption performances have also been reported when Cd(II) ions were used as template ions [143] (Table 5). The Cd(II)-imprinted aerogels displayed an excellent selectivity for Cd(II) ions when they were studied in aqueous mixtures with Pb(II, Co(II), Zn(II), Hg(II), Ni(II), Fe(III), or NH_4_^+^ ions. The values of K_d_ and K_Cd/M_ of the ion-imprinted aerogel were significantly higher in comparison to those of the non-imprinted one [143].

Polymer-modified magnetic nanoparticles have also received great attention as efficient sorbents for removal of several metal ions (Cu(II), Co(II), Ni(II), Zn(II), Hg(II), etc.) due to the easy separation of the spent sorbent by an external magnetic field [144,147]. Thus, arginine functionalized magnetic CS beads were successfully used for simultaneous removal of Cu(II), Co(II), and Ni(II) ions from aqueous solution [147]. The sorption kinetics were well fitted by the PSO model, while the sorption isotherm data obeyed the Freundlich model. The reusability study indicated the efficacy of the composite beads in four sorption/desorption cycles determining the sorption capacity was preserved by up to 70%. Sorbents endowed with a stable performance during sorption/desorption cycles have been developed by Benettayeb et al. [144]. Magnetic glycine-*g*-CS microparticles were synthesized and investigated for removal of hazardous and strategic metal ions from tailing leachates. The composite microparticles were successfully used for the recovery of Hg(II), Ni(II), and Zn(II) from single component solutions. The elution of the HMIs adsorbed on the composite sorbent was completely achieved with acidic urea solution. This sorbent was reused for three cycles with no significant loss in sorption performance. The composite microparticles were also tested for the treatment of acidic leachates from Egyptian tailing ore. It was found that the composite sorbent recovered a wide panel of HMIs, with preference for Zn(II), Ni(II), and Y(III) since they were in a high concentration in the feed solution. However, this sorbent was poorly selective and adsorbed many of the metal ions present in the leachates.

Magnetic CS nanocomposites modified with GO and PEI have also been prepared and applied as sorbents for removal of both HMIs, such as As(III), Hg(II), and anionic azo-dyes (CR and Amaranth), from aqueous solution [164]. The maximum adsorption capacities of nanocomposites for As(III), Hg(II), CR, and Amaranth were 220.26 mg/g, 124.84 mg/g, 162.07 mg/g, and 93.81 mg/g, respectively. The sorbent exhibited a good stability in acidic solution with no significant difference in the adsorption capacity during five sorption/desorption cycles only for CR, Amaranth, and As(III). In the case of Hg(II) sorption, the efficacy of the sorbent decreased to about 60% after the fifth cycle of sorption [164].

In another approach, magnetic CS microparticles grafted with amidoxime or hydrazinyl amine have been developed and tested for recovery of U(VI) and Zr(IV) from aqueous solutions [145]. FTIR and XPS analysis demonstrated the chemical modifications and the contribution of amidoxime and hydrazinyl amine moieties in the binding of metal ions through a tautomerization effect. The sorption equilibrium was relatively fast, attained in less than 60 min, and the uptake kinetics were efficiently described by the PFO model. The maximum sorption capacities depended on the type of the metal ion and the sorbent functional groups; thus, 328.44 mg U(VI)/g was sorbed by amidoxime-functionalized composite and 178.36 mg Zr(IV)/g was retained by hydrazinyl-modified composite. The desorption of the metal ions was successfully achieved with 0.3 mol/L HCl solution and the regenerated sorbent was efficient for five cycles of sorption and desorption. However, after the fifth cycle a progressive decrease in the sorption performance was observed. The evaluation of selectivity from bi-component solutions showed that amidoxime-modified composite was selective for U(VI) over Zr(IV), especially at pH 4, while the hydrazinyl-modified composite equally adsorbed the two metal ions. The application of the sorbents to the treatment of ore leachates confirmed the selectivity of amidoxime-modified magnetic CS microparticles for U(VI) [145].

#### 3.4.2. Organic/Inorganic Composites for Dyes Sorption

Since the environmental pollution with non-biodegradable and toxic dyes occurs all over the world, the development of environmentally friendly composite sorbents has lately received a growing interest. Numerous organic–inorganic composite sorbents, consisting of polysaccharides (CS, ALG, CEL, kCG, Pul), clays (such as bentonite and MMT) [150,152,153,154,155], zeolites [141,151], GO [156,157,158,164], or magnetic nanoparticles [161,163] have been recently developed and tested for removal of cationic dyes. For example, MB has been efficiently removed by sorption on porous composite hydrogels prepared by intercalation of MMT nanosheets within CMC/CS systems [155]. It was found that the Sips isotherm model well fitted the experimental equilibrium data and the maximum sorption capacity for MB was 283.9 mg MB/g sorbent (Table 5). The composite sorbents have presented an excellent level of reusability, with no major changes in the sorption performance even after five consecutive sorption/desorption cycles. High sorption capacity for MB has also been reported for ALG/CPL composite beads [151]. The ALG/CPL beads exhibited an improved sorption capacity for MB (452.25 mg/g) in comparison to ALG beads (151.73 mg/g) and CPL (48.12 mg/g). The desorption of MB from the ALG/CPL composites was achieved with 0.1 M CH_3_COOH solution. The reusability studies showed a slight decrease in the sorption capacity of ALG/CPL sorbent after the third cycle of sorption/desorption. Ahmadi et al. [161] have reported the preparation of MMT/St/CoFe_2_O_4_ nanocomposites as efficient sorbents for MV and MB dyes from synthetic and real wastewater. The impact of various parameters (such as pH, temperature, initial dye concentration, contact time, and sorbent dose) on the dye decontamination has been optimized with the response surface-central composite design method. The ability of the MMT/St/CoFe_2_O_4_ sorbent to treat textile industrial wastewater was tested under optimized conditions. Thus, this sorbent had the capability to decrease the ADMI value from 950 to 245 (i.e., a removal efficiency of 74.21%). In addition, the BOD5, COD, and TOC values after wastewater treatment with MMT/St/CoFe_2_O_4_ sorbent have been reduced by 86.92, 77.23, and 70.77%, respectively [161]. Malachite green (MG), an organic cationic dye widely utilized in the textile and paper industries, has been successfully removed by sorption on porous ALG-*g*-PAA/graphite hydrogel composites synthesized by graft polymerization of acrylic acid onto ALG and subsequent loading with graphite powder [160]. The sorption process was well described by the Langmuir isotherm model and PSO kinetic model. Its sorption capacity for MG reached 628.93 mg/g at pH 7 in 1h. The sorption/desorption study indicated a good level of reusability of the hydrogel composites with a removal efficiency of 91% and 79.63% after the third and fifth cycle, respectively [160].

CV is another cationic dye which has been efficiently removed by sorption on polydopamine (PDA)/MMT/Pul composite hydrogels [154]. The equilibrium sorption data were best fitted by the Langmuir isotherm and the maximum sorption capacity for CV was 112.45 mg CV/g sorbent. The effectiveness of these composite sorbents has also been evaluated in real water samples, namely river water (Oujiang, China) and local industrial wastewater (Wenzhou, China). It was found that the PDA/MMT-containing Pul hydrogel maintained its high CV sorption capacity in river water, whereas in the industrial wastewater the sorption capacity of the composite sorbent decreased to 82.64 mg/g [154]. XG*-g*-(PAA-*co*-PAMPS)/GO composite hydrogels have also been recently reported as remarkable sorbents for CV with a maximum theoretical sorption capacity of 1566.97 mg/g [159]. This composite sorbent has exhibited excellent selectivity in separation of CV from its mixture with MO. In addition, it has a very high level of reusability (>95%) with no changes in the sorption capacity observed after twenty consecutive cycles of sorption/desorption. The column sorption tests performed with this composite hydrogel further confirmed its huge potential to treat large quantities of CV-containing wastewater [159]. A novel magnetic nanocomposite based on CPL, St, and CoFe_2_O_4_ has been synthesized by the co-precipitation method and successfully used in the removal of MB, MV, and CV from aqueous media [162]. The CPL/St/CoFe_2_O_4_ magnetic adsorbent exhibited excellent sorption performance in removal of all dyes at an initial pH of 9. The equilibrium sorption data were in good agreement with Langmuir and Redlich–Peterson isotherm models. In addition, this sorbent was successfully used to treat textile industry wastewater, where the value of the ADMI parameter was diminished from 938 to 224, which means a removal efficiency of 76.12% [162]. Recyclable and efficient adsorbents for removal of MB, neutral red (NR), and Safranine T (ST) have also been prepared by employing GO modified Fe_3_O_4_ as MNP-doped ALG/CS gel microspheres. The sorption kinetics fitted the PFO model, indicating a sorption mechanism driven by van der Waals interactions, hydrogen bonds, and electrostatic forces. The reusability studies showed a removal rate higher than 90% even after five consecutive sorption/desorption cycles, supporting further practical applications of magnetic gel microbeads.

A simple one-step chemical process has been reported by Yu et al. [163] for the preparation of aminated magnetic CS microspheres (TETA-MCTSms) as efficient sorbents for removal of an anionic dye, namely reactive brilliant red (RBR). The magnetic CS microspheres (MCTSms) were further modified with PMAA to design sorbents capable of additionally removing cationic dyes such as MB. TETA-MCTSms sorbent exhibited remarkable sorption properties for RBR, with a maximum sorption capacity of 637.41 mg/g at pH 2, whereas the MCTSms-PMAA sorbent showed an excellent sorption performance for MB, represented by a maximum sorption capacity of 211.22 mg/g at pH 12. The X-ray photoelectron spectroscopy analysis indicated a mechanism of dye sorption mainly driven by electrostatic interactions and hydrogen bonding [163].

#### 3.4.3. Organic/Inorganic Composite Hydrogels for the Sorption of Oxyanions

Beside HMIs and dyes, other contaminants such as phosphate [165], dichromate (Cr_2_O_7_^2−^) [167], or chromate (HCrO_4_^−^, CrO_4_^2−^) [166,168] have been successfully removed by different organic–inorganic composites. For example, Wang et al. have developed an eco-friendly lanthanum cross-linked PVA/ALG/palygorskite hydrogel composite for phosphate removal from aqueous solutions [165].

**Figure 12:**
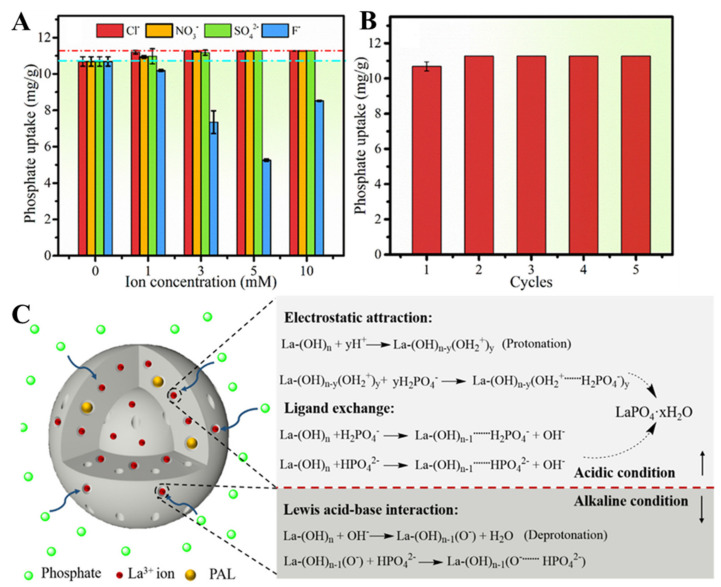
Influence of co-existing ions on phosphate uptake (**A**), sustainability upon consecutive sorption/desorption cycles (**B**), and the possible phosphate removal mechanism (**C**) by PVA/ALG/palygorskite composite beads (Reprinted with permission from Ref. [165]. Copyright 2023 Elsevier.)

The maximum sorption capacity reached at pH 4 was 32.2 mg H_2_PO_4_^−^/g sorbent. An efficient phosphate removal (nearly 100%) was also maintained in the presence of Cl^−^, NO_3_^−^, and SO_4_^2−^ as coexisting anions (Figure 12A). The sorption process was satisfactorily fitted by the Freundlich isotherm model and PSO kinetic model. Thermodynamic studies showed that the sorption process was spontaneous and endothermic. The generated composite sorbent was reused for five cycles without any loss of the initial sorption capacity (Figure 12B). The sorption mechanisms proposed by the authors based on FTIR, XRD, and XPS results were electrostatic interactions and ligand exchange in an acidic environment, whereas under alkaline conditions Lewis acid–base interactions prevailed (Figure 12C) [165].

In another work, porous biochar/ALG/PEI composite beads prepared by He et al. have also been successfully tested for removal of Cr(VI) from aqueous media [166]. A maximum sorption capacity of 769.2 mg/g was achieved at pH 2.0. Moreover, these composite beads were applied to treat highly concentrated electroplating wastewater from the Jiangyan Longgou Electroplating Factory [166]. The concentration of Cr(VI) from the electroplating complex mixtures was reduced to below 0.05 ppm when a sorbent dose of 1.4 g/L was used. However, the sorption capacity of the composite beads decreased to about 50% after the fourth cycle of adsorption. For removal of various species of Cr(VI) from aqueous solution, including Cr_2_O_7_^2−^, CrO_4_^−^, and CrO_4_^2−^, CS has been either modified with zirconium-based MOFs (MOF-808) [167], graphene oxide [167], or carbon nanotubes and iron [174]. According to the Langmuir model, MOF-808/CS composites exhibited, at pH 5, a maximum Cr(VI) sorption capacity of 320.0 mg/g1. The adsorption of Cr(VI) in the presence of some co-existent anions, such as PO_4_^3−^, SO_4_^2−^, NO_3_^−^ and Cl^−^, was also investigated. It was found that the adsorption capacity of Cr(VI) ions by MOF-808/CS sorbent composites was drastically reduced in the presence of competing anions, following the order: PO_4_^3−^ > SO_4_^2−^ > NO_3_^−^ > Cl^−^, which was associated with the charge density order of these anions. Density functional theory (DFT) calculations were applied to study the interaction energy and reduced density gradient of Cr(VI) ion adsorption on MOF-808/CS composites. The DFT results indicated that the Cr_2_O_7_^2−^ anions, as the main form of Cr(VI), adsorbed onto these composites through hydrogen bonds and electrostatic interactions [167]. Kong et al. [168] developed GO/CS composite aerogels cross-linked with 3-glycidoxypropyltrimethoxysilane by combining sol–gel methodology with freeze-drying. The GO/CS aerogels exhibited remarkable stability in acidic pH and high performance in removal of Cr(VI) ions. The adsorption capacity of GO/CS composite aerogels for Cr(VI) was kept high even in the presence of some other ions, such as Cu(II), Cd(II), Cr(III), SO_4_^2−^, or PO_4_^3−^. The GO/CS aerogels showed good reusability, with the removal efficiency decreasing to about 58% after the fifth cycle of adsorption. Recently, an interesting study on Cr(VI) removal using a continuous-flow column packed with CS/carbon nanotubes/iron composite beads has been reported by Aslam et al. [174]. A maximum of 54% of Cr(VI) ion removal was achieved at a feed concentration of 30 mg/L, a flow rate of 1 mL/min, and a bed height of 8 cm. In addition, the key mass transfer parameters were determined with the homogeneous surface diffusion model. The calculated axial dispersion and diffusion coefficients varied in the range between 10^−8^ and 10^−7^ m^2^/s, and 10^−11^ and 10^−10^ m^2^/s^−1^, respectively. Furthermore, it was shown that the column adsorption efficiency can be improved at higher bed heights and lower flow rates and feed concentrations.

## 4. Considerations on the Use of Polysaccharides as Building Blocks to Prepare Sustainable Materials for Wastewater Treatment

The selection of suitable polysaccharides for preparing sustainable composite materials is performed considering their economic competitiveness, processability, efficiency, and functionality [17]. The extraction/purification technologies of polysaccharides from corresponding raw materials have significantly improved over the years; nowadays most of them are commercialized at accessible prices. Exceptions to this are the glycosaminoglycans, such HA and CRS (Figure 2), that are basic components of the extracellular matrix in vertebrates. As a result of their elevated costs, as well as their poor mechanical properties and rapid hydrolysis, HA and CRS are not envisaged as sustainable raw materials for preparing composite sorbents for wastewater treatment [175,176]. However, they have been extensively used in cutting-edge healthcare applications, including the development of soft hydrogels for osteoarthritis treatment, cartilage repair, ophthalmology, skin rejuvenation, peripheral nerve regeneration, and drug delivery [177,178,179].

Most polysaccharides depicted in Figure 2 are soluble in water, this being a significant advantage in developing affordable and environmental-friendly sorbents. Albeit their widespread availability and low cost, Cel and CT are, however, the only two exceptions, because of a dense network of intra-/intermolecular hydrogen bonds. Thus, their utilization is limited only to nanofillers aiming to improve the mechanical properties of various composites. Simple and facile chemical transformations of Cel and CT that yield water-soluble derivatives easier to be processed into multifunctional materials have been developed over the years. As example, by deacetylation (alkaline, enzymatic, or ultrasonic) of CT, CS, a biopolymer soluble in acidic aqueous media, is produced [180]. Prompted by the high chemical reactivity of the free amino groups, CS offers plentiful opportunities to develop innovative materials for energy, healthcare, or environmental applications [181,182]. Particularly, for water purification, CS stands out as a highly sustainable raw material in fabricating numerous low-cost composite sorbents with remarkable mechanical properties, chemical stabilities, and sorption performances [181]. On the other hand, the chemical modification of Cel by grafting different multifunctional ligands or monomers is extensively studied worldwide as a means to produce soluble derivatives [183], aiming to broaden the use of Cel-based materials in many advanced applications [184,185,186].

In the frame of wastewater treatment, the adsorption efficiency of prepared materials strongly depends on the number and availability of functional groups containing electron-rich atoms. The native wealth of hydroxyl, amino, carboxyl, and sulfate groups in polysaccharides promotes them as valuable alternatives to synthetic polymers for adsorption of HMIs, dyes, oxyanions, or pesticides from contaminated waters [181,182,186,187]. Besides their inherent capacity to interact with different pollutants, polysaccharides can also support different chemical modifications, either as a path to generate stable networks (cross-linking), or as a means to graft new multifunctional ligands to augment their sorption performance. For example, CS, the only cationic polysaccharide, can easily react with aldehydes to yield condensates known as Schiff bases. When CS is reacted with dialdehydes (such as GA), cross-linked hydrogel networks are produced [29,187]. Other reactions at the amino groups, including alkylation, thiolation, or phosphorylation, can also be performed to obtain valuable CS derivatives [188,189,190]. Anionic polysaccharides (HA, CRS, ALG, XG, and Pec) can also undergo specific chemical reactions at the carboxyl groups, including esterification and amidation [191]. The glycosidic units of all polysaccharides contain multiple hydroxyl groups; therefore, their acylation, phosphorylation, or chlorosulfonation are also important in synthesizing new multifunctional derivatives [191,192].

Although beneficial in terms of sorption performance, the feasibility of such reactions should also be analyzed with respect to energy and environmental impacts. Desirably, such reactions should be performed in “green” solvents (such as water) and at ambient temperatures, they should proceed quickly, be quantitative, and should not yield toxic side products [193]. Deviations from these conditions directly reflect in the cost of final products and/or their environmental and ecological fingerprints. However, the use of organic solvents or elevated temperatures cannot always be circumvented [189,194]. Careful considerations on whether certain reactions are appropriate in the context of the current quest for “green” solutions to well-known pollution issues, without creating new ones, are thus mandatory.

## 5. Conclusions and Perspectives

**Figure 13:**
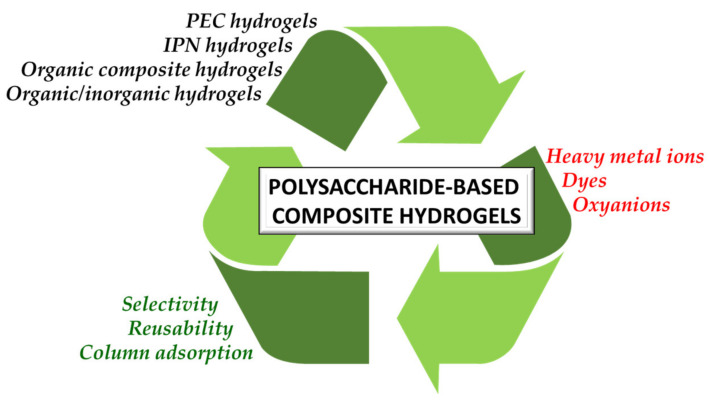
Illustration summarizing the types of polysaccharide-based composite hydrogels and their application in wastewater treatment from a sustainability perspective.

Adsorption is envisaged worldwide as a highly efficient technology to tackle the growing problems associated with water pollution, an exhaustive up to date portfolio of materials has thus been developed that remove contaminants (HMIs, dyes, oxyanions, pharmaceutic compounds, nitrophenols) from wastewater. Hydrogels containing at least one polysaccharide in their composition are a distinct class of sorbents that exhibit many unique advantages derived from their bioavailability, low cost, and structure of polysaccharides. In this context, here we reviewed the recent literature available on polysaccharide-based organic and organic/inorganic composite hydrogels for HMI, dye, and oxyanion removal from wastewater. The emerging picture is that all developed polysaccharide composites (Table 2, Table 3, Table 4 and Table 5) show remarkable sorption capacity for all types of pollutants. However, their performance is strongly influenced by several characteristics, including sorbent dose, ionic strength, temperature, pH, and medium composition (content of each pollutant in the aqueous solution, presence of competing cross-contaminants, etc.). The sustainability of these materials in wastewater treatment applications is currently focused on three main directions: selectivity, reusability, and column adsorption (Figure 13).

However, more attention should be paid to production and operation costs, and also to their environmental fingerprints. Thus, focus should be directed to simple preparation strategies that can produce resilient and versatile composites with abundant and accessible sorption sites. The designed materials should be mechanically and chemically stable, and also capable of quickly retaining high amounts of pollutants. The cost factor and inherent environmental impact should be carefully scrutinized upon selecting the best adsorption system. Column processes are considered more economically and environmentally advantageous; thus, more attention should be paid to pollutant sorption in dynamic conditions as a translational stage towards pilot scale or even industrial level application.

Composites possess a finite number of interaction sites; therefore, the main approach to increase their economic feasibility is to elute the sorbed pollutants and reuse the composites in multiple sorption/desorption cycles. However, this creates large volumes of concentrated waste that are highly detrimental to the environment. Alternative desorption pathways and valorization strategies of spent sorbents should be further investigated to reduce the environmental fingerprint of composite hydrogels for wastewater treatment applications. For example, the “turning waste into treasure” concept is currently attracting attention as a sustainable means to achieve a so-called circular economy. Thus, prospective valorization strategies of spent sorbents should be followed in order to minimize their economic and environmental impact.

## Figures and Tables

**Table 1:** Common models for fitting the experimental sorption data (isotherms, kinetic and column).

Fitting Models	Nonlinear	Linear	Parameters
Isotherms			
Langmuir	$q_{e}=\frac{q_{m}K_{L}C_{e}}{1+K_{L}C_{e}}$	$\frac{C_{e}}{q_{e}}=\frac{C_{e}}{q_{m}}+\frac{1}{K_{L}q_{m}}$	*q_e_* (mg/g) = sorbed amount at equilibrium; *C_e_* (mg/L) = equilibrium concentration; *q_m_* (mg/g) = maximum sorbed amount. *K_L_* (L/mg) = Langmuir adsorption constant; *K_F_* = Freundlich constant; *1/n* = parameter related to surface heterogeneity; *a_S_* (L/mg) = Sips adsorption constant.
Freundlich	$q_{e}=K_{F}C_{e}^{1/n}$	$lnq_{e}=lnK_{F}+\frac{1}{n}lnC_{e}$	
Sips	$q_{e}=\frac{a_{S}C_{e}^{1/n}}{1+a_{S}C_{e}^{1/n}}$	$\frac{1}{n}lnC_{e}=-ln\left(\frac{a_{S}}{q_{e}}\right)+lna_{S}$	
Kinetics			
Pseudo-first-order (PFO)	$q_{t}=q_{e}\left(1-e^{-k_{1}t}\right)$	$ln\left(q_{e}-q_{t}\right)=lnq_{e}-k_{1}t$	*q_e_* (mg/g) = sorbed amount at equilibrium; *qt* (mg/g) = sorbed amount at time *t*; *k_1_* (min^−1^) = PFO model rate constant; *k_2_* (g/mg∙min) = PSO model rate constant; *t* (min) = time.
Pseudo-second-order (PSO)	$q_{t}=\frac{k_{2}q_{e}^{2}t}{1+k_{2}q_{e}t}$	$\frac{1}{q_{t}}=\frac{1}{k_{2}q_{e}^{2}}+\frac{1}{q_{e}}$	
Column experiments			
Thomas	$\frac{C_{t}}{C_{0}}=\frac{1}{1+exp\left[\left(\frac{k_{TH}q_{0}m}{Q}\right)-k_{TH}C_{0}t\right]}$	*ln(C_0_/C_t_–1) = (k_TH_∙q_0_∙m)/Q-k_TH_∙C_0_∙t*	*C_0_* (mg/L) = initial concentration; *C_t_* (mg/L) = concentration at time *t*; *C_e_* (mg/L) = equilibrium concentration; *q_0_* (mg/g) = maximum sorption capacity; *Q* (mL/min) = flow rate. *k_TH_* (L/mg∙min) = Thomas rate constant; *k_YN_* (mL/mg∙min) = Yoon–Nelson rate constant; *τ* (min) = time required to reach 50% breakthrough; *t* (min) = time.
Yoon–Nelson	$\frac{C_{t}}{C_{0}}=\frac{1}{1+exp\left[\left(k_{YN}\left(\tau -t\right)\right)\right]}$	*ln[C_e_/(C_0_–C_e_)] = k_YN_∙t-τ∙k_YN_*	

**Table 2:** HMIs sorption performance by some PEC hydrogels.

PEC hydrogels	HMI	q_max_, mg/g	Comments	Ref.
SL/LCS	Ni(II)	411.8	Batch; Monocomponent; Parameters: pH 7, ≈ 1h, SL/LCS ratio = 7/3; Fitting: Langmuir/ PSO; Reusability: >95% (5 cycles).	[73]
SL/CMCS	Pb(II)	415.6	Batch; Monocomponent/Mixture; Parameters: pH 6, 1h, SL/CMCS ratio = 6/4; Fitting: Langmuir/ PSO; Selective for Pb(II) in mixture with Fe(III), Cr(III), Cd(II), Cu(II), Zn(II), Ni(II), Hg(II) and Co(II); Reusability: >95% (5 cycles).	[74]
CS/ALG/Ca^2+^	Pb(II) Cu(II) Cd(II)	176.50 70.83 81.25	Batch; Monocomponent; Parameters: pH 5, 298 K; Fitting: Sips/PSO.	[75]

Abbreviations: CMCS—carboxymethyl-CS; LCS—lactate-CS.

**Table 3:** Pollutants sorption performance by semi-IPN/IPN composite hydrogels.

Semi-IPN/IPN Hydrogels	Pollutants	q_max_, mg/g	Comments	Ref.
EDTA-*g*-CS/PAAm DN hydrogel	Cd(II) Cu(II) Pb(II)	86.00 99.44 138.41	Batch; Monocomponent/Mixture; Parameters: pH 5, 6h, 298 K, dose 1 g/L; Fitting: Langmuir/PSO; Selectivity: Cu(II) > Pb(II) > Ni(II) > Cd(II) > Zn(II) > Mn(II); Reusability: 94.1% (5 cycles).	[43]
PAAm/St-*g*-PAA semi-IPN hydrogel	Cu(II) Cd(II) Ni(II) Zn(II)	40.72 19.27 9.31 7.48	Batch; Monocomponent; Parameters: pH 4.7, 24h, T = 295 K; Fitting: Sips.	[76]
CS-*g*-PAA/Gel semi-IPN hydrogel	Cu(II)	261.08	Batch; Monocomponent; Parameters: pH 5.75, 1h, 293 K, dose 2 g/L; Fitting: Langmuir/PSO; Reusability: 95.2% (5 cycles).	[77]
Pec/P(AAm-*co*-AGA) semi-IPN hydrogel	Cu(II) Ni(II)	203.7 121.7	Batch; Monocomponent/Mixture; Parameters: pH 5, 24h, 341 K, dose 0.2 g/L; Fitting: Langmuir/PSO; Selectivity: Ni(II) > Cu(II) > Co(II) Reusability: 92% (5 cycles).	[78]
CMC/PAAm semi-IPN hydrogel	Cu(II) Pb(II) Cd(II)	227.3 312.5 256.4	Batch; Monocomponent/Mixture; Parameters: pH 5.5, 24 h, 298K, dose 0.5 g/L; Fitting: Langmuir/PSO; Reusability: 90.9% (3 cycles); Application in catalytic reduction of 4-nitrophenol.	[79]
α-ketoglutaric acid-*g*-CS/PAAm semi-IPN hydrogel	Cu(II) Pb(II) Zn(II)	72.39 51.89 61.41	Batch; Monocomponent/Mixture; Parameters: pH 5, 4 h, 303 K, dose 1 g/L; Fitting: Langmuir/PSO; Selectivity: Cu(II) > Pb(II) > Zn(II) > Ni(II) > Cd(II) Reusability: 90% (5 cycles).	[80]
XG/PAA/Cloisite15A semi-IPN hydrogel	Co(II) Cu(II) Ni(II)	436.62 530.14 511.74	Batch; Monocomponent; Parameters: 24 h, 298 K; Fitting: Temkin/PSO; Reusability: 30% (5 cycles).	[81]
Lignin/CS/PAAm IPN hydrogel	Pb(II) Cu(II) Cd(II)	374.32 196.68 268.98	Batch; Monocomponent/Mixture; Parameters: pH 5, 2h, 298 K, dose 1 g/L; Fitting: Freundlich/PSO; Selectivity: preferential sorption of Pb(II), Cu(II) and Cd(II) over Zn(II), Ni(II) and Mn(II) ions; Reusability: 100% (5 cycles).	[82]
St/PAA IPN hydrogel	Cd(II)	256.4	Batch/Column; Monocomponent/Mixture; Parameters: pH 5, 4h, 298 K, dose 1 g/L; Fitting: Langmuir/PSO; Selectivity: preferential sorption for Pb(II) and Cd(II) over Zn(II), Mn(II), Ni(II) and Cu(II) ions; Reusability: 97.7% (5 cycles).	[42]
ALG/PAA/GO DN hydrogel	Cd(II)	115.65	Batch; Monocomponent/Mixture; Parameters: pH 6, 12h, 313 K, dose 1 g/L; Fitting: Langmuir/PFO; Selectivity: Pb(II) > Cu(II) > Cd(II) > Mn(II) Reusability: 40% (5 cycles).	[44]
ALG-*g*-PAA/PVA semi-IPN hydrogel	Pb(II)	784.97	Batch; Monocomponent; Parameters: pH 5, 2 h; 303 K, dose 2 g/L; Fitting: Langmuir/PSO; Reusability: 93.6% (5 cycles).	[83]
GG/XG/PAA semi IPN hydrogel	Pb(II) Hg(II)	111.6 86.4	Batch; Monocomponent Parameters: pH 5, 24 h, 300 K, dose 1 g/L.	[84]
Pul-*g*-PAAm semi-IPN hydrogel	Hg(II)	1725	Batch; Monocomponent/Mixture; Parameters: 1.5 h, 292 K, dose 0.01 g; Fitting: Freundlich/PSO; Selectivity: minor change of Hg(II) sorbed amount in the presence of Ca(II), Fe(III), Mg(II) and Zn(II); Reusability: 86% (3 cycles).	[85]
CS/PAAm IPN cryogels	MB	750	Batch; Monocomponent/Mixture; Parameters: pH 5.5, 24 h, 298 K, dose 1 g/L; Fitting: Sips/PFO; Selective sorbent for MB in its mixture with MO; Reusability: 100% (4 cycles).	[86]
PAA-*g*-St/PAAm semi-IPN cryogels	MB	443.7	Batch; Monocomponent; Parameters: pH 6.5, 24 h, 298 K, dose 1 g/L; Fitting: Sips/PFO; Reusability: 100% (6 cycles).	[87]
PAA-*g*-St/PAA semi-IPN cryogels	MB	667.7	Batch; Monocomponent; Parameters: pH 6.5, 24 h, 298 K, dose 1 g/L; Fitting: Sips/PFO; Reusability: 100% (6 cycles).	[87]
ALG/P(AAm-*co*-AA) semi-IPN hydrogel	BF MV	763 550	Batch; Monocomponent; Parameters: pH 7, 24 h, 298 K; Fitting: Langmuir /PSO; Reusability: 98% (5 cycles).	[88]
St/P(AAm-*co*-HEMA) semi-IPN hydrogel	MG MV	388 315	Batch; Monocomponent; Parameters: pH 7, 24 h, 298 K, dose 5 g/L; Fitting: Langmuir /PSO; Reusability: 98.5% (5 cycles).	[89]
PAA/PVA/yeast IPN hydrogel	MB	629	Batch; Monocomponent/Mixture; Parameters: pH 8, 24 h, 303 K, dose 1 g/L; Fitting: Langmuir/PSO; Selectivity: MB > ST > MO > AF at pH 10 Reusability: 81.5% (5 cycles).	[90]
CS/PEG-*co*-AAm semi-IPN hydrogel	AR18 AO7 MO	342.54 221 185.24	Batch; Monocomponent; Parameters: pH 2, 24 h, 298 K, dose 0.6 g/L; Fitting: Langmuir/PSO.	[91]
CS/St semi-IPN hydrogel	DR80	340.86	Batch; Monocomponent/Mixture; Parameters: pH 3, 5 h, 323 K, dose 0.5 g/L; Fitting: Freundlich/PSO; DR80 sorbed amount decreased in the presence of competing anions: Cl^−^ < NO_3_^−^ < SO_4_^2−^ <PO_4_^3−^; Reusability: 75% (4 cycles).	[92]
PDMAEMA/CMCS IPN hydrogel	ST IC	126 130.5	Batch; Monocomponent/Mixture; Parameters: pH 3, 72 h, 298K, dose 1.5 g/L; Fitting: Langmuir/PFO; Selective for ST at pH > 3; Simultaneous removal of ST and IC at pH 3; Reusability: 93% (3 cycles).	[93]
Zr-loaded magnetic CS/PVA IPN hydrogel	H_2_PO_4_^−^	54.08	Batch; Monocomponent/Mixture; Parameters: pH 5, 24 h, dose 0.25 g/L, 309 K; Fitting: Langmuir/PFO; Selectivity: Small decrease of H_2_PO_4_^−^ sorbed amount in the presence of humic acid, Cl^−^, HCO_3_^−^, NO_3_^−^ or SO_4_^2−^; Reusability: 96% (5 cycles).	[94]
CS/PEI IPN hydrogel	H_2_PO_4_^−^	343	Batch; Monocomponent/Mixture; Parameters: pH 3, 298 K, dose 0.75 g/L; Fitting: Langmuir/PSO; Selectivity: Low influence from competing anions (Cl^−^, NO_3_^−^, SO_4_^2−^); Reusability: 92% (5 cycles).	[95]
CS/PEI/PDMAEMA IPN sponges	H_2_PO_4_^−^	408.89	Batch; Monocomponent/Mixture; Parameters: pH 4, 24 h, 296 K, dose 0.75 g/L; Fitting: Sips/PSO; Selectivity: Low influence from competing anions (Cl^−^, NO_3_^−^, SO_4_^2−^); Reusability: 95% (5 cycles).	[39]

Abbreviations: AO7—Acid Orange 7; AF—Acid fuchsin; AR18—Acid Red 18; BF—basic fuchsin; DR80—Direct Red 80; EDTA—ethylenediaminetetra-acetic acid; HEMA—hydroxyethyl methacrylate; IC—Indigo carmin; MB—methylene blue; MG—malachite green; MV—methyl violet; PAA—poly(acrylic acid); PAAm—poly(acrylamide); P(AAm-*co*-AGA)—poly(acrylamide-*co*-acrylamidoglycolic acid); PDMAEMA—poly [2-(dimethylamino)ethyl methacrylate]; PEG—poly(ethyene glycol); PEI—poly(ethyleneimine); PVA—poly(vinyl alcohol); ST—Safranine T.

**Table 4:** Pollutant sorption performance by different polysaccharide-based composite hydrogels.

Organic Composite Hydrogels	Pollutants	q_max,_ (mg/g)	Comments	Ref.
CS/St-*g*-PAN cryobeads	Cu(II) Ni(II) Co(II)	100.6 83.25 74.01	Batch; Monocomponent; Parameters: pH 5 for Cu(II) and pH 6 for Ni(II) and Co(II), dose 1 g/L, 300 K; Fitting: Langmuir/PSO; Reusability: 95% (5 cycles).	[106]
CS/St-*g*-PAMOX beads	Cu(II)	238.14	Batch; Monocomponent; Parameters: pH 4.5, dose 5 g/L, 298 K; Fitting: Langmuir/PSO; Reusability: >98% (5 cycles).	[23]
Glucan/CS hydrogels	Cu(II) Co(II) Ni(II) Pb(II) Cd(II)	342 232 184 395 269	Batch; Monocomponent; Parameters: pH 7, 3 h, 293 K; Fitting: Langmuir/PSO.	[27]
Sulfonate modified CS/Arabic gum biosorbent	U(VI)	471.24	Batch; Monocomponent/Mixture; Parameters: pH 4, 20 min, 325 K; Fitting: Sips/PFO; Selective for U(VI) in mixture with Fe(III), Cu(II), Zn(II), Mg (II), Al(III) and Ca(II) at pH > 4.17; Reusability: 97% (5 cycles).	[107]
P(AA-*co*-DMAEMA)/CS aerogels	Cu(II)	131.6	Batch; Monocomponent; Parameters: pH 6, 8 h, 298 K; Fitting: Langmuir/PSO; Desorption performed by CO_2_ bubbling => no side products; Reusability: 70% (6 cycles).	[108]
CEL/CS aerogels	Cu(II)	260.41	Batch/Column; Monocomponent/Mixture; Parameters: Cu(II)–pH 6 and 250 min, CR–pH 8 and 800 min; Fitting: Langmuir /PSO/Thomas/Yoon–Nelson; Synergetic influence of preadsorbed CR on Cu(II) sorption, and vice versa.	[109]
	CR	380.23		
ALG/PEI hydrogels	Cu(II) Pb(II)	322.6 344.8	Batch; Monocomponent/Mixture; Parameters: pH 5.5, 7 h, 298 K; Fitting: Langmuir/PSO; Application in catalytic reduction of nitrophenols.	[110]
PVA/ALG hydrogel beads	Pb(II)	139.37	Batch; Monocomponent/Mixture; Parameters: pH 5 for Pb(II) and pH 3 for Cr(VI), 6 h, 298 K; Fitting models: Langmuir /PSO; Tested in electroplating, municipal and river wastewaters; Reusability: 85% (5 cycles).	[111]
PVA/ALG/CS hydrogel beads	Cr(VI)	86.14		
ALG/XG beads–freeze dried	MB	545.6	Batch/Column; Monocomponent; Parameters: pH 7, 298 K; Reusability: 87% (4 cycles).	[112]
SL-*g*-(AAm-*co*-sodium allylsulfonate)	RhB	71.6	Batch; Monocomponent; Parameters: pH 7, 298 K; Fitting: Langmuir/ PSO; Reusability: 85.76% (5 cycles)	[113]
kCG/PGMA hydrogel beads	MB	166.62	Batch; Monocomponent; Parameters: pH 7, 4 h; Fitting: Langmuir/PSO; Reusability: 83.3% (5 cycles).	[114]
PANI/ALG	MB RhB Orange-II MO	555.5 434.78 476.19 146.66	Batch; Monocomponent/Mixtures; Parameters: pH 3 (Orange-II and MO) and pH 9 (MB and RhB), 4 h, 308 K; Fitting: Langmuir /PSO; Selective for anionic dyes (MO and Orange-II) at pH 3 and for cationic dyes (MB and RhB) at pH 9.	[115]
TA/PVA/ALG hydrogel beads	MB	147.06	Batch; Monocomponent; Parameters: pH 10, 12 h, 303 K; Fitting: Langmuir/PSO; Reusability: 81% (5 cycles).	[116]
HESt/P(APTMACl) hydrogels	MV MO	6.62 238.1	Batch; Monocomponent/Mixture; Parameters: pH > 6, 24 h, 298 K; Fitting: Langmuir/PFO; Selectivity: HESt/P(APTMACl) for anionic dyes, and HESt/PAA for cationic dyes.	[117]
HESt/PAA hydrogels	MV MO	185.2 2.84		
HESt/PAAm hydrogels	MV MO	9.17 3.33		
CEL-*g*-P(AA-*co*-PAAm) biosorbents	MB Acid blue 93	1372 1372	Batch; Monocomponent/Mixture; Parameters: pH 7, 80 min, 498 K; Fitting: Freundlich/PSO; Selectivity: moderate sorption performance loss in binary dye systems, and in the presence of small counterions and surfactants; Reusability: 85% (3 cycles).	[118]
XG-*g*-P(AMPS-*co*-AAm)	MB	384.62	Batch; Monocomponent; Parameters: pH 7, 3 h, 298 K; Fitting: Langmuir/PSO; Reusability: 83.5% (6 cycles).	[119]
St-*g*-PAA	MB	2967.66	Batch; Monocomponent; Parameters: pH 9, 6 h, 308 K; Fitting: Langmuir/PSO; Reusability: 72% (4 cycles).	[120]
CS/PVAm/IEx cryobeads	Cr(VI)	317.94	Batch; Monocomponent; Parameters: pH 5.5, dose 1.25 g/L, 200 min; Fitting: Langmuir /PFO; Reusability: >60% (5 cycles).	[121]
PEI/CMC hollow beads	Cr(VI) H_2_PO_4_^−^	535.39 150.65	Batch/Column; Monocomponent/Mixture; Parameters: Cr(VI): pH 2, 400 min; H_2_PO_4_^−^: pH 3, 200 min; Fitting: Langmuir /PFO/Thomas; Selectivity: phosphate sorption significantly decreased in the presence of F^−^ and SO_4_^2−^; Reusability: Cr(VI)–6 cycles; H_2_PO_4_^−^–22 cycles.	[122]
CMC/CMCS hydrogels	H_2_PO_4_^−^	93.5	Batch; Monocomponent/Mixture; Parameters: pH 2, 6 h, 298 K; Fitting: PSO; Selectivity: influence of competing ions followed the order Cl^−^ < NO_3_^−^ < SO_4_^2−^.	[123]
CEL/PEI aerogels	Cr(VI)	229.1	Batch/Column; Monocomponent/Mixture; Parameters: pH 2, flow rate: 1 to 3 mL/min; Fitting: Freundlich/PSO; Selectivity: PO_4_^3−^ and SiO_3_^2−^ significantly reduced Cr(VI) sorption performance; Reusability: >80% (5 cycles).	[124]
NaLS/PEI/ALG beads	Cr(VI)	2500	Batch/Column; Monocomponent/Mixture; Parameters: pH 2, 6 h, 298 K; Fitting: Langmuir/PSO; Selectivity: minimum influence from humic acids and coexisting anions; Applied in secondary electroplating wastewater treatment in columns.	[125]

Abbreviations: HESt—hidroxyethyl starch; IEx—ion exchange resins; kCG–k-carrageenan; NaLS—sodium lignosulfonate; PAMOX—poly(amidoxime); P(AMPS-*co*-AAm)—poly(2-acrylamido-2-methyl propane sulfonic acid)-*co*-acrylamide); PAN—polyacrylonitrile; PANI—polyaniline; P(APTMACl)—poly(3-(acrylamidopropyl) trimethyl ammonium chloride); PGMA—poly(glycidyl methacrylate); PVAm—polyvinylamine; RhB—rhodamine B; TA—tanic acid.

**Table 5:** The pollutants sorption performance by different organic/inorganic composite hydrogels.

Organic/Inorganic Composite Hydrogels	Pollutants	q_max_, (mg/g)	Comments	Ref.
CS/MMT beads	Cu(II) Ni(II) Pb(II) Zn(II)	13.04 12.18 29.85 13.50	Batch; Monocomponent/Mixture; Parameters: pH 8, 1 h, 298 K; Fitting: Freundlich, Langmuir/PSO; Selectivity: Pb(II) > Cu(II) > Zn(II) > Ni(II).	[137]
Magnetic bentonite-CS beads	Cs(I)	57.1	Batch; Monocomponent/Mixture; Parameters: pH 8.5, 8 h, 298 K; Fitting: Langmuir/ PSO; Selective for Cs(I) in its mixture with Li(I), Na(I), K(I) and Mg(II); Reusability: 95–100% (5 cycles).	[138]
P(AMOX)-g-CS/bentonite composite	U(VI)	49.09	Batch; Monocomponent/Mixture; Parameters: pH 8, 1 h, 303 K; Fitting: Langmuir/ PSO; Selectivity: U(VI) >> Pb(II) >> Cd(II) >> Cu(II) > Ni(II) >> Fe(III); Reusability: 95% (6 cycles).	[139]
CS_EDTA_-CPL cryobeads	Co(II), Zn(II), Cd(II), Pb(II), Ni(II)	145.55	Column; Mixture; Parameters: pH 4.5, Flow rate = 1 mL/min; Fitting: Thomas/Yoon–Nelson; Selectivity: Ni(II) > Pb(II) ≥ Cd(II) > Zn(II) > Co(II).	[29]
ALG/biochar beads	La(III) Ce(III) Pr(III) Nd(III)	88.03 123.95 125.53 107.14	Batch; Monocomponent; Parameters: pH 5, 8 h, 293 K; Fitting: Langmuir/PSO; Reusability: 97% (6 cycles).	[140]
ALG/CPL beads	La(III) Ce(III) Pr (III) Nd(III)	18.04 30.58 34.44 19.33	Batch; Monocomponent; Parameters: pH 5, 8 h, 293 K; Fitting: Langmuir/ PSO; Reusability: 97% (6 cycles).	[140]
XG-Glutathione/Zeolite nanocomposites	Pb(II) Ni(II) CR	42.91 47.98 40	Batch; Monocomponent; Parameters: pH 4 for Pb(II), 5 for Ni(II) and 2.1 for CR, 2 h for Pb(II) and Ni(II)) and 4 h for CR, 293 K; Fitting: Langmuir/PSO; Reusability: 60% (5 cycles).	[141]
Ion imprinted SL/GO sponges	Hg(II)	413.6	Batch; Monocomponent/Mixture; Parameters: pH 7, 1 h, 293 K; F itting: Freundlich/PSO; Selective for Hg(II) in its mixture with Ni(II), Co(II), Cu(II), Pb(II), Cd(II) and Zn(II); Reusability: 95% (5 cycles).	[142]
Ion imprinted SL/GO aerogels	Cd(II)	412.5	Batch; Monocomponent/Mixture; Parameters: pH 6–8, 1 h, 293 K; Fitting: Langmuir/PSO; Selective for Cd(II) in its mixture with Cu(II), Pb(II), Co(II), Zn(II), Hg(II), Ni(II), Fe(III) and NH_4_^+^; Reusability: >95% (5 cycles).	[143]
Fe_3_O_4_/Glycine-modified CS composites	Ni(II) Zn(II) Hg(II)	29.5 30.55 70.0	Batch; Monocomponent/Mixture; Parameters: pH 5–6, 8 h, 294 K; Fitting: Langmuir/PSO; Selectivity: Hg(II) >> Pb(II) >> Cu(II) >> Ni(II) >> Zn(II); Reusability: 98% (3 cycles).	[144]
Fe_3_O_4_/CS-*g*-AMOX microparticles	U(VI)	328.44	Batch; Monocomponent/Mixture; Parameters: pH 3, 40 min; Fitting: Freundlich/PFO; Selective for U(VI) over Zr(IV) at pH 4 Reusability: 96% (5 cycles); Tested in real ore leachate wastewater.	[145]
Fe_3_O_4_/CS-*g*-hydrazinyl amine microparticles	Zr(IV)	178.36	Batch; Monocomponent/Mixture; Parameters: pH 4, 1 h; Fitting: Freundlich /PFO; Selectivity: equally binds U(VI) and Zr(IV); Reusability: 97% (5 cycles).	[145]
Magnetic Xanthate-Modified CS/PAA Hydrogels	Cu(II) Cd(II) Pb(II) Co(II)	206 178 168 140	Batch; Monocomponent; Parameters: pH 5.5, 4 h, 303 K; Fitting: Langmuir, Freundlich/PFO; Reusability: >50% (10 cycles).	[146]
Arginine functionalized Fe_3_O_4_/CS beads	Cu(II), Co(II) Ni(II)	172.4 161.2 103.0	Batch; Monocomponent; Parameters: pH 6, 160 min, 303 K; Fitting: Freundlich/PSO; Reusability: 70% (4 cycles).	[147]
γ-MnO_2_/CS_EDTA_/Fe_3_O_4_ nanocomposites	Zn(II) Pb(II)	310.4 136.0	Batch; Monocomponent/Mixture; Parameters: pH 6, 2 h, 293 K; Fitting models: Langmuir/PSO; Selectivity: simultaneous removal of Zn(II) and Pb(II); Reusability: 85% (8 cycles).	[148]
PEI/CS/α-MnO_2_ foams	U(VI)	301.9	Batch; Monocomponent/Mixture; Parameters: pH 4.5, 2 h, 298 K; Fitting: Langmuir/PSO; Selective for U(VI) in the presence of Th(IV), Eu(III), Fe(III), Al(III), Co(II), Pb(II), Ni(II) or Cu(II); Reusability: 87% (5 cycles).	[149]
ALG/bentonite/biochar beads	MB	47.39	Batch; Monocomponent; Parameters: pH 6–8, 2 h, 303 K; Fitting: Langmuir/PSO.	[150]
ALG/CPL beads	MB	452.25	Batch; Monocomponent; Parameters: pH 5.5, 6 h, 298 K; Fitting: Langmuir/PSO; Reusability: 100% (3 cycles).	[151]
CEL/clay/ALG composites	MB	38.00	Batch; Monocomponent; Parameters: pH 11, 1 h, 303 K; Fitting: Freundlich/PSO.	[152]
CMC/kCG/MMT beads	MB	12.25	Batch; Monocomponent; Parameters: pH 6–10, 2 h, 303 K; Fitting: Langmuir/PSO; Reusability: 95% (5 cycles).	[153]
Polydopamine/MMT/Pul hydrogel composites	CV	112.45	Batch; Monocomponent/Mixture; Parameters: 6 h, 310 K; Fitting: Langmuir/PSO; Selective for cationic dyes (CV, MB); Reusability: 95% (4 cycles).	[154]
CMC/CS/TiO_2_@MMT	MB	283.9	Batch; Monocomponent; Parameters: pH 8, 2 h, 298 K; Fitting: Sips/PFO and PSO; Reusability: 95% (5 cycles).	[155]
ALG/GO beads	MB	12.64	Batch; Monocomponent; Parameters: pH 7.78, 12 h, 298 K; Fitting: Freundlich.	[156]
St/GO composites	MB	500.0	Batch; Monocomponent; Parameters: pH 7, 4 h, 318 K; Fitting: Freundlich.	[157]
ALG/GO@Fe_3_O_4_/CS beads	MB NR ST	21.32 44.65 44.31	Batch; Monocomponent; Parameters: pH 7, 2 h; Fitting: PFO; Reusability: >90% (5 cycles).	[158]
XG-*g*-(PAA-*co*-PAMPS)/GO hydrogel composites	CV	1566.97	Batch; Monocomponent/Mixture; Parameters: pH 7, 1 h, 298 K; Fitting: Langmuir/PSO; Selective for CV over MO; Reusability: >95% (20 cycles).	[159]
ALG-*g*-PAA/graphite porous hydrogels	MG	628.93	Batch; Monocomponent; Parameters: pH 7, 1 h, 298 K; Fitting: Langmuir/PSO; Reusability: 91% (3 cycles).	[160]
MMT/St/CoFe_2_O_4_ nanocomposites	MV MB	43.95 47.51	Batch; Monocomponent; Parameters: pH 6, 40 min, 318 K; Fitting: Langmuir, Redlich-Peterson/PFO; Reusability: 70% (8 cycles).	[161]
CPL/St/CoFe_2_O_4_	CV MB MV	32.84 31.81 31.15	Batch; Monocomponent; Parameters: pH 9, 1 h, 303 K; Fitting: Langmuir, Redlich-Peterson/PFO; Reusability: 70% (10 cycles).	[162]
Aminated Fe_3_O_4_/CS microspheres	RBR	549.39	Batch; Monocomponent; Parameters: pH 7, 2 h, 293 K; Fitting: Langmuir/PFO.	[163]
Fe_3_O_4_/CS-*g*-PMAA microspheres	MB	103.9	Batch; Monocomponent; Parameters: pH 10, 2h, 293 K; Fitting: Langmuir/PFO.	
Magnetic CS/GO/PEI nanocomposites	As(III) Hg(II) CR Amaranth	220.26 124.84 162.07 93.81	Batch; Monocomponent; Fitting: Langmuir /PSO; Reusability: 60–95% depends on pollutant (5 cycles).	[164]
PVA/ALG/palygorskite beads	H_2_PO_4_^−^	33.2	Batch; Monocomponent/Mixture; Parameters: pH 4, 6 h, 313 K; Fitting: Freundlich/PSO; Selectivity in the presence of co-existing anions (Cl^−^, NO_3_^−^, SO_4_^2−^F^−^); Reusability: 100% (5 cycles).	[165]
Biochar-ALG/PEI composite beads	HCrO_4_^−^	769.2	Batch; Monocomponent; Parameters: pH 2, 24 h, 298 K; Fitting: Langmuir/PSO; Reusability: 50% (6 cycles); Tests on Cr(VI) removal from electroplating wastewater.	[166]
MOF-808/CS composites	Cr_2_O_7_^2−^	320.00	Batch; Monocomponent; Parameters: pH 5, 30 min, 298 K; Fitting: Langmuir/PSO; Reusability: 72% (6 cycles); Selectivity in the presence of PO_4_^3−^, SO_4_^2^, NO_3_^−^ and Cl^−^ anions.	[167]
GO/CS composite aerogels	HCrO_4_^−^ Cr_2_O_7_^2−^	146	Batch; Monocomponent; Parameters: pH 2.5, 120 min, 298 K; Fitting: Langmuir/PSO; Reusability: 58% (5 cycles); Selectivity in the presence of interfering ions: Cu(II), Cd(II), Cr(III), SO_4_^2−^, or PO_4_^3−^.	[168]

Abbreviations: CPL—clinoptilolite; CV—crystal violet; NR—neutral red; MMT—montmorilonite.

## Data Availability

Not applicable.
